# Role of water and protein dynamics in proton pumping by respiratory complex I

**DOI:** 10.1038/s41598-017-07930-1

**Published:** 2017-08-10

**Authors:** Outi Haapanen, Vivek Sharma

**Affiliations:** 1 Department of Physics, University of Helsinki, P. O. Box 64, FI-00014 Helsinki, Finland; 2 Department of Physics, Tampere University of Technology, P. O. Box 692, FI-33101 Tampere, Finland; 3 Institute of Biotechnology, University of Helsinki, Helsinki, Finland

## Abstract

Membrane bound respiratory complex I is the key enzyme in the respiratory chains of bacteria and mitochondria, and couples the reduction of quinone to the pumping of protons across the membrane. Recently solved crystal or electron microscopy structures of bacterial and mitochondrial complexes have provided significant insights into the electron and proton transfer pathways. However, due to large spatial separation between the electron and proton transfer routes, the molecular mechanism of coupling remains unclear. Here, based on atomistic molecular dynamics simulations performed on the entire structure of complex I from *Thermus thermophilus*, we studied the hydration of the quinone-binding site and the membrane-bound subunits. The data from simulations show rapid diffusion of water molecules in the protein interior, and formation of hydrated regions in the three antiporter-type subunits. An unexpected water-protein based connectivity between the middle of the Q-tunnel and the fourth proton channel is also observed. The protonation-state dependent dynamics of key acidic residues in the Nqo8 subunit suggest that the latter may be linked to redox-coupled proton pumping in complex I. We propose that in complex I the proton and electron transfer paths are not entirely separate, instead the nature of coupling may in part be ‘direct’.

## Introduction

Complex I (NADH-quinone oxidoreductase), the largest enzyme in the respiratory chains of eukaryotes and several bacteria, serves as a redox-linked reversible proton pump^1^. It catalyzes the reduction of quinone (Q) by NADH, and couples the reaction to pumping of protons across the membrane^2^. The proton electrochemical gradient drives the ATP-synthase to convert ADP and P_i_ into ATP^3^. For detailed structural, functional and mechanistic aspects of complex I, see refs 4–8.

**Figure 1:**
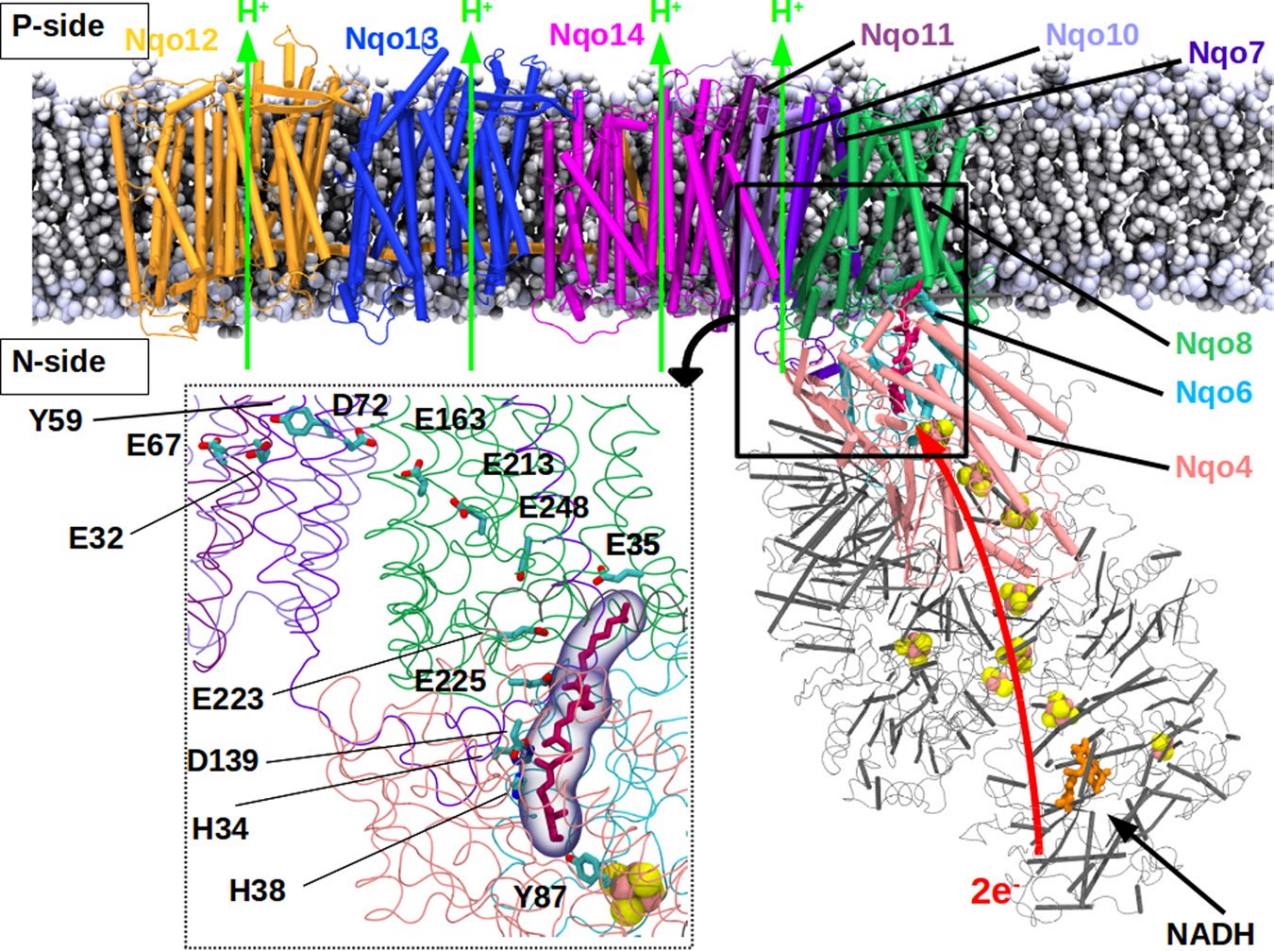
Structure of respiratory complex I embedded in lipid-solvent environment. The membrane-embedded antiporter-type subunits Nqo12–14 are shown in orange, blue and magenta, respectively. Nqo8 (green), Nqo7, Nqo10 and Nqo11 (violet-indigo) are also displayed along with the hydrophilic subunits Nqo6 (light blue) and Nqo4 (pink) that form the Q-binding site scaffold. Q (purple) binds in a buried binding site. All other hydrophilic subunits are shown in grey. Covalently-bound FeS clusters (pink-yellow spheres), and FMN (orange) at the terminal end of the hydrophilic domain are also shown. NADH binds next to FMN and acts as a direct donor of two electrons to the latter. The region studied in this work is marked by a thick black box, and is shown to atomic details in the inset (box with dotted lines). The residues discussed in this work are also marked (inset).

Complex I consists of two domains; a membrane-bound hydrophobic domain and a hydrophilic domain, which resides in the mitochondrial matrix or bacterial cytoplasm (also N-side, see Fig. 1). NADH binds on the edge of the hydrophilic domain and within pi-stacking distance from the non-covalently bound flavin mono nucleotide FMN (Fig. 1). The electrons released upon NADH oxidation are first transferred to FMN, and subsequently to Q ca. 100 Å away, via a chain of covalently bound FeS clusters (Fig. 1). Based on electron transfer theory^9^, and kinetic experiments^10, 11^, the rate of electron transfer from NADH to Q has been found to be ca. 100 µs. Biochemical experiments^12–14^ and crystal or cryo-EM structures^15–21^ have provided significant insights into how Q may bind close to the N2 FeS cluster in a long tunnel-like cavity. In one possible arrangement the head group of Q is in hydrogen bonding contact with the conserved and functionally critical Tyr87 of Nqo4 subunit^18^. For complex I subunit nomenclature, see Supplementary Table S1. All amino acid numbering corresponds to the respiratory complex I from *Thermus thermophilus*, unless otherwise stated.

The seven membrane-bound hydrophobic subunits (Nqo7, Nqo8, Nqo10–14) are thought to be involved in proton translocation (see Fig. 1). The three subunits Nqo12–14 share homology with the Na^+^/H^+^ antiporters^22, 23^, and various site-directed mutagenesis studies support their role in proton pumping^24–28^. The three smaller membrane-bound subunits (Nqo7, Nqo10–11), together with Nqo8, provide the connectivity between the membrane arm and the hydrophilic domain of the enzyme (Fig. 1). Among these subunits Nqo8 has been proposed to be involved in proton translocation based on X-ray structure analysis^18^.

The coupling mechanism between the electron transfer and proton pumping is of significant interest because the latter occurs at a maximal distance of 200 Å from the electron transfer path, and is three orders of magnitude slower. To this date, the molecular understanding of the coupling has stayed elusive, but it has been suggested that it is the reduction of the bound Q that drives the proton pump via conformational changes supplemented by electrostatics^18, 19, 29–32^. See also refs 4–8 for various aspects associated with complex I mechanism.

It was recently proposed that the two-electron reduction of bound-Q at the site close to the N2 FeS cluster occurs simultaneously with the abstraction of two protons; one from the conserved Tyr87, and another one from the His38/Asp139 ion pair, in the Nqo4 subunit^30^. Moreover, it was observed in the simulations that large scale conformational changes occur in the Q-binding site and in the Nqo8 subunit^30^. In response to the dissociation of Asp139 from neutral His38, the latter partially stabilizes the deprotonated Tyr^30^, which is in agreement with conformation observed in the crystal structure^19^. It has also been argued that there are two Q-binding sites in complex I, and the “crystallographic” Q after reduction from N2 further reduces a second loosely-bound Q, which then exchanges with an oxidized quinone from the membrane^2, 33^. Alternatively, there is a single Q-binding site close to the N2 center, and a Q molecule reduced at the latter site is released into the lipid membrane in exchange for an oxidized Q, thereby completing the turnover^4, 34^. Nevertheless, it remains entirely unclear how the reactions that take place in the Q-tunnel communicate with the distant proton pumping subunits. Here, we investigate this question by performing fully-atomistic classical molecular dynamics (MD) simulations on the entire structure of complex I from *Thermus thermophilus* in lipid-solvent environment. Based on simulation data, we suggest that charged amino acid residues in Nqo8 and Nqo7 subunits undergo protonation-state dependent conformational changes, which are important for redox-coupled proton pumping in complex I. The simulation data highlight the key role of protein hydration in this long-range coupling between the Q-binding site and the antiporter-type subunits. Moreover, we find that premature protonation of a key residue of Nqo4 subunit (Tyr87) near the Q-binding site may be effectively prevented by electrostatic stabilization from a conserved arginine from Nqo6. The mechanistic insights from our work are crucial in understanding the causes behind various mitochondrial disorders that may occur due to malfunction in one of the many steps of the catalytic cycle of the enzyme.

## Results

### Hydration and dynamics of antiporter-like subunits

Fully atomistic classical MD simulations performed on the entire crystal structure of complex I show that the root mean square deviation (RMSD) of protein stabilizes at a value of 4–5 Å in the first 50–100 ns of simulation time (Supplementary Fig. S1). The RMSD of the membrane domain, comprising Nqo7, Nqo8, Nqo10–14 subunits, also converges to a stable value of ca. 4 Å within the same time domain. The data suggests that the system is fully equilibrated, and timescales of simulation are sufficient to analyze fast dynamics associated with the protein and the water molecules.

**Figure 2:**
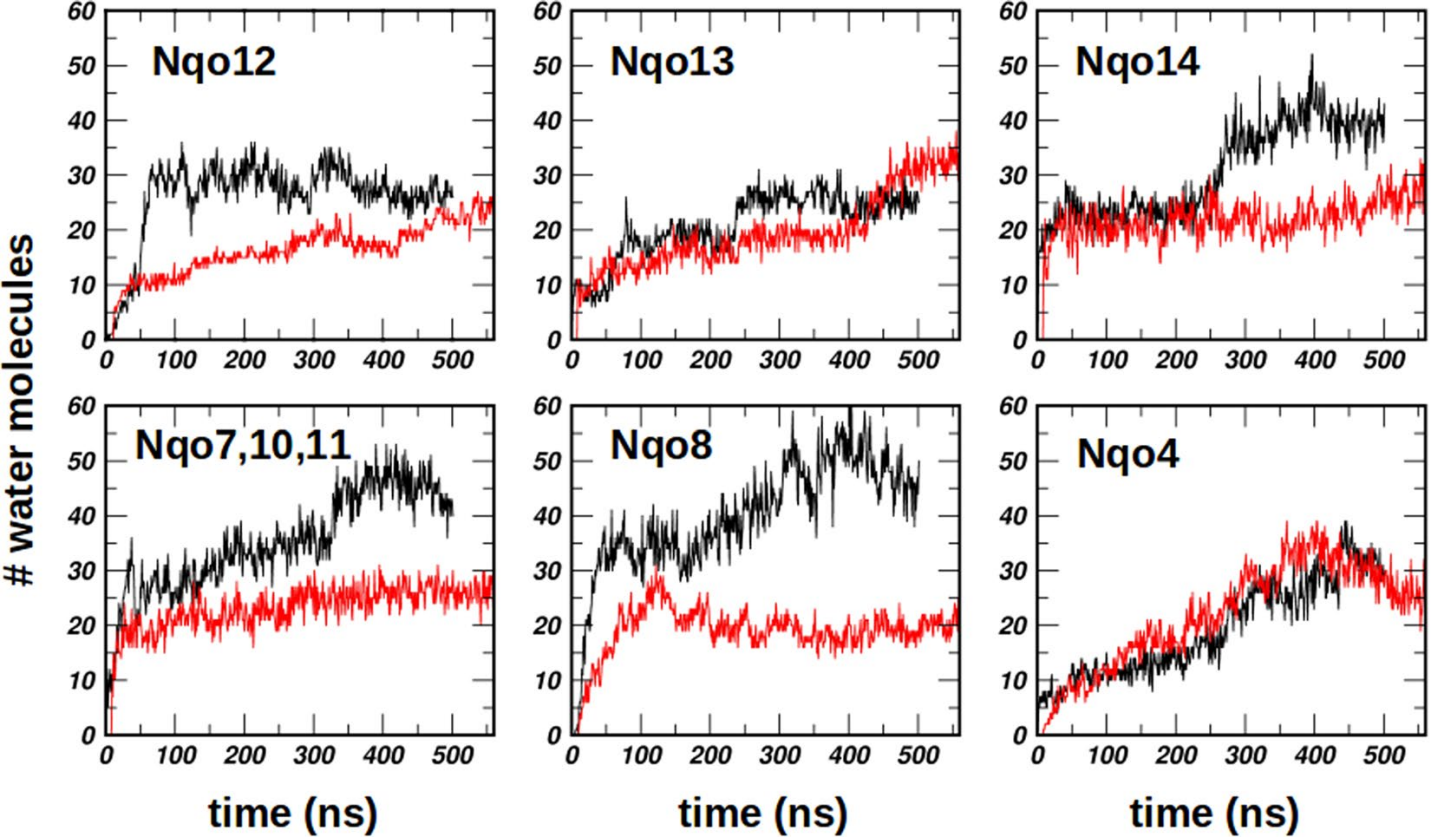
Hydration of the protein subunits based on simulations of setup I (black) and setup II (red) with Q^ox^ and QH_2_, respectively (see also Table 1). Number of water molecules is plotted (ordinate) *vs* the simulation time (abscissa, in ns). Only water molecules (oxygen atom) within 4 Å of the selected amino acids are considered in the analysis (see Supplementary Table S1).

Starting from a completely dehydrated protein, the two 500 ns simulation trajectories (setups I and II) reveal rapid hydration of the protein that is within the first 100 ns (Fig. 2). The three membrane-bound subunits Nqo12–14, which are homologous to the Na^+^/H^+^ antiporters, are found to hydrate extensively (Fig. 2). In each of these subunits, the water molecules enter from both sides of the membrane, and a sigmoidal shaped pathway is transiently formed, which is in agreement with the earlier structural and simulation studies^18, 19, 31, 35^.

After an initial steep rise, the hydration in different membrane bound subunits plateau to relatively stable values in the rest of the simulation time (100–500 ns). However, some short term fluctuations in water occupancy are observed in Nqo7/10/11 and Nqo8 subunits occurring at around 400 ns (see black curves in the lower panel of Fig. 2). These occur due to transient hydration and dehydration events in a particular region of the protein. This is more clearly displayed in Supplementary Fig. S2, which shows that the maximum change in hydration (dehydration) at around 400 ns takes place next to certain specific residues (Asn35 of Nqo10, Asn36 of Nqo11, and Glu213 and Glu35 of Nqo8). Similarly, the increase in hydration in Nqo13 (setup II) and in Nqo14 (setup I) at around 400 and 250 ns, respectively (Fig. 2), occurs partly due to the flipping of the sidechains of amino acid residues. The sidechain flip of His211 (Nqo13) and Lys216 (Nqo14), shown as a change in RMSD and sidechain dihedral in Supplementary Figs S2 and S3, respectively, coincides with the increase in hydration in the nearby region (see also Supplementary Table S1). Since, sidechain fluctuations are likely to occur in these simulation timescales, we suggest that the hydration in the membrane-bound subunits is stable, and small variations occur around an equilibrium value.

**Figure 3:**
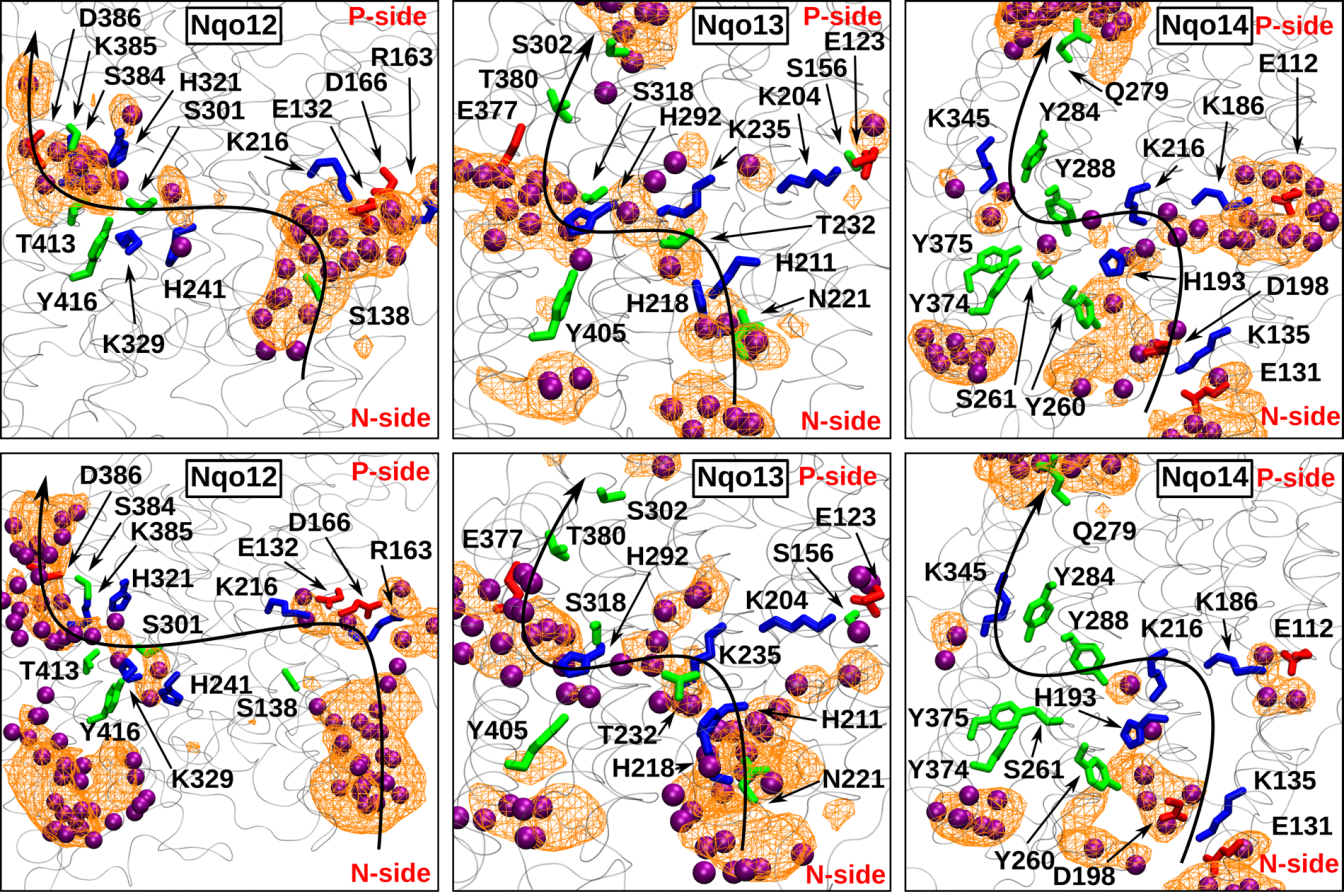
Proton channels in antiporter-type subunits Nqo12–14 from simulation setups I (top) and II (bottom) with Q^ox^ and QH_2_, respectively (see also Table 1). The putative proton transfer paths from the N-side to the P-side are marked with an S-shaped black arrow. The residues that take part in water-wire formation are also listed (acidic – red, basic – blue, and polar - green). Water molecules are shown in purple. The water positions are instantaneous, and represent a simulation snapshot. The water occupancy, calculated by averaging water positions over entire simulation trajectory, is displayed as an orange mesh plotted with an isovalue of 0.1. Water molecules within 6 Å of selected residues (Supplementary Table S1) were used to calculate the occupancy plot.

In subunit Nqo12, the water-based connection between the middle of the subunit and the P-side of the membrane (‘P-path’) forms readily in both 500 ns MD simulations I and II, and comprises conserved charged or polar amino acid residues (Fig. 3 and Supplementary S4). In addition to the putative proton exit route or the P-path, two proton uptake paths are observed (‘N-path’), one closer to the interface between the Nqo12 and Nqo13 subunits in setup II, and the other one enclosed in subunit Nqo12 in setup I (Fig. 3, see also Supplementary Fig. S4). The interfacial hydration close to the N-side of the membrane was also observed in the earlier simulation study by Kaila *et al*. on *E. coli* enzyme^31^. However, in contrast to that our simulation data show substantial hydration confined to Nqo12 subunit (upper panel in Fig. 3 from simulation of setup I) that is around the conserved residue Ser138 (Supplementary Table S1). In our simulations, a transient connectivity is also observed between the two paths (N and P-paths) formed by water molecules and His241 of Nqo12 (Fig. 3). However, the water occupancy in this middle region remains low (Fig. 3 and Supplementary S4), and may form a barrier preventing proton leak from the P-side to the N-side of the membrane. Almost all of the Nqo12 residues that participate in proton channel formation are found to be conserved in the *E. coli* enzyme (Supplementary Table S1), and the observed hydration in this region is also in good agreement with the earlier predictions from X-ray and simulation data^17, 18, 31^.

In order to further elucidate the role of water molecules in proton pumping by complex I, we analyzed the electric dipole moment of water molecules in various hydrated regions of the protein (Supplementary Fig. S5). Remarkably, the total electric dipole moment vector of water molecules orients in such a way that would allow efficient transfer of protons from the N-phase of the membrane to the conserved proton acceptor, Glu132, in the middle of the Nqo12 subunit (see panel A in Supplementary Fig. S5). The water dipole and hydrogen bond arrangements observed further corroborate the proton transfer pathways identified in this work.

During simulations of setups I and II we observed stabilization of the ion-pair formed by two highly conserved residues, Arg163 of Nqo12 and Glu377 of neighboring Nqo13 subunit, which tightens the subunit-subunit interface (Supplementary Fig. S6). In various crystal or cryo-EM structures, the distance between the two residues (Arg:Cζ – Glu:Cδ distance) is 6.3 Å^18^, 9.6 Å^19^, 8.5 Å^20^ and 8.1 Å^21^, whereas this distance reduces to ca. 4.3 Å in the two longest simulations (setups I and II) due to the ion-pairing. Moreover, this ion-pair is placed in a particularly interesting location in partly non-polar surroundings in the middle of the membrane (Supplementary Fig. S6). Due to the presence of charged residues (see also Supplementary Table S2), the region gets rapidly hydrated and water molecules are exchanged between the two subunits. The stabilized subunit-subunit interface may play a role in the long-range coupling between the Q-reduction and proton pumping in distant subunit Nqo12.

In contrast to Nqo12, rather large number of water molecules are found to diffuse in the middle of subunits Nqo13 and Nqo14 (Fig. 3 and Supplementary S4), most likely due to the presence of charged residues Lys235 and Lys216 of Nqo13 and Nqo14, respectively (*cf*. neutral His241 in Nqo12 in Supplementary Table S2). A closer look to the hydration in the middle of the subunits Nqo13 and Nqo14 reveal preferred orientation of water dipoles that is favorable for Grotthuss-type proton transfer^36^ (Supplementary Fig. S5). The water-based connectivity between the middle of the subunit and the N-side of the membrane is also established in both the subunits (Fig. 3). However, the hydration close to the P-side of the membrane is found to be low in both the simulation setups I and II (see also Supplementary Fig. S4). The relatively low water occupancy in this region may represent a conformation in which proton ‘back-leak’ is minimized.

The two residues (Lys235 in Nqo13, and Lys216 in Nqo14) that are seen to participate in proton channel formation in our simulations are conserved in the *E. coli* complex I (Lys265 in NuoM and Lys247 in NuoN, respectively, see Supplementary Table S1). These residues were also found to attract water molecules in their vicinity in the earlier simulation study by Kaila *et al*. (ref. 31). Overall, in agreement with earlier work, our simulation data reveal high and low water occupancies in the regions close to the N- and the P-side of the membrane, respectively.

In stark contrast to the Nqo12/13 subunit-subunit interface (Supplementary Fig. S6), the hydration of Nqo13/14 interface is not observed, primarily due to the missing inter-subunit ion pairing. However, despite this the interface is stabilized by the formation of transient H-bonds in the loops of Nqo13 and Nqo14 subunits (Supplementary Fig. S7). Similar stabilization is also observed for the Nqo12/13 and Nqo14/7/10 interfaces suggesting that in addition to the long horizontal helix, loop-loop interactions may also keep antiporter-type subunits together.

Even though several similarities are observed between the two longest simulations (setups I and II), some differences are also apparent. First is the presence of water molecules in the lower part of Nqo12 subunit in simulation of setup II (lower panel of Fig. 3). However, when hydration data from other setups are analyzed (Supplementary Fig. S4), virtually there is no water occupancy in this region of the enzyme, suggesting that the observed hydration is transient, and is probably due to the stochastic nature of the simulations. Similarly, when data from other simulations are taken into account (Supplementary Fig. S4), sufficient water density is found in the middle of the Nqo14 subunit, similar to Nqo13. Overall, the sequence and structural homology between antiporter-type subunits, partial overlap of water distribution in the three subunits (Fig. 3 and Supplementary S4) as well as similarity with the earlier simulation work on *E. coli* enzyme (see Supplementary Table S1), further strengthen our results and conclusions.

### Fourth proton pathway

**Figure 4:**
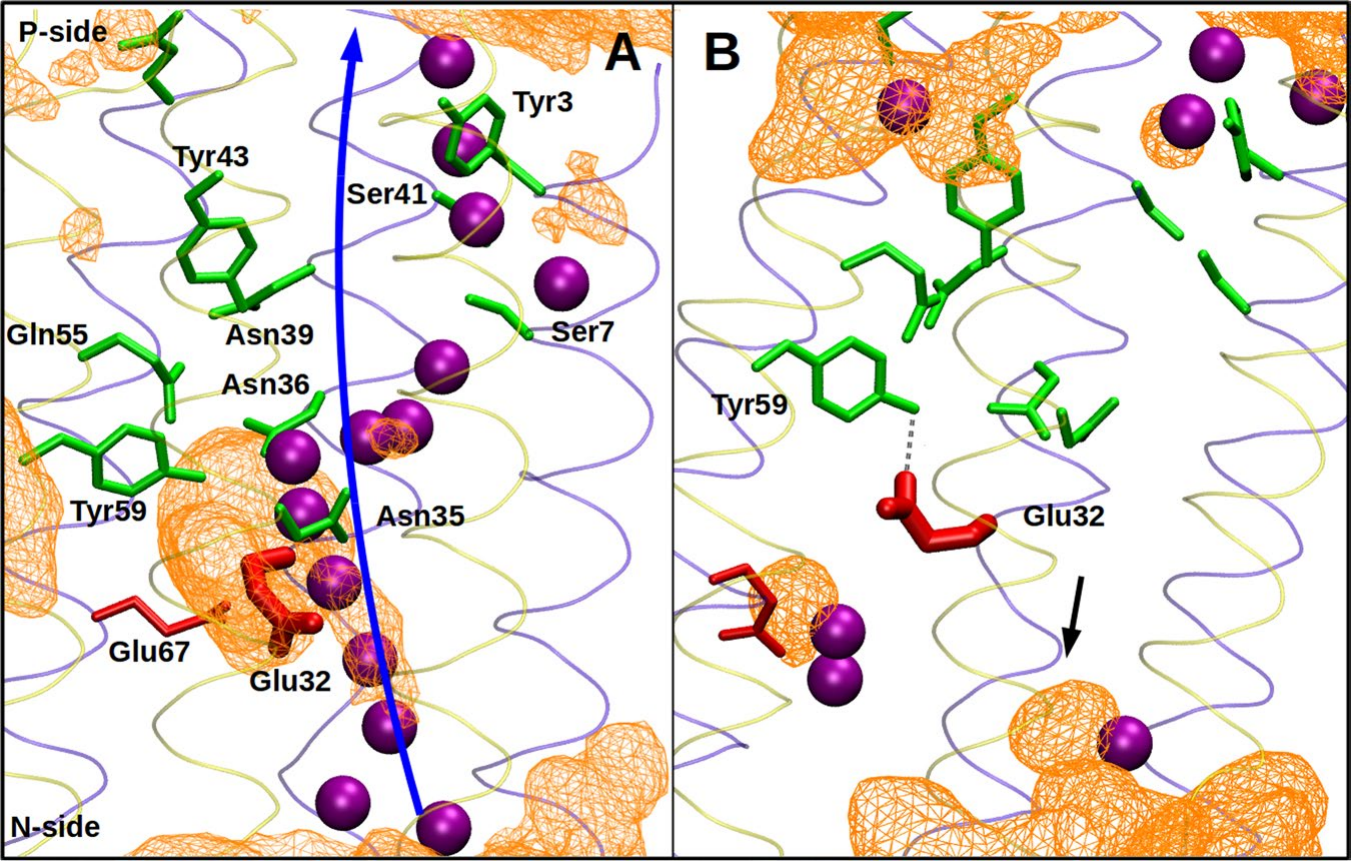
Fourth putative proton pathway at the interface of Nqo10 (yellow ribbons) and Nqo11 (blue ribbons). The water occupancy map at a surface isovalue of ca. 0.1 is displayed with an orange colored wireframe. The functionally important Glu32 is shown in thick licorice representation in its down flipped ((**A**) simulation setup I) and crystallographic ((**B**) simulation setup II) conformations. Water molecules are shown as purple spheres, and other polar or charged residues in green and red, respectively. Hydrogens are omitted for clarity. The figure is prepared from simulation setups I (**A**) and II (**B**), and water occupancy is calculated by counting water molecules within 5 Å of the polar residues of helices in Nqo10 and Nqo11 subunits. The H-bond between Tyr59 and Glu32 is shown with a grey dotted line, and the arrow in panel B highlights the water density near Glu32.

Consistent with the presence of three homologous subunits (Nqo12–14) is the proton pumping stoichiometry of 3 H^+^/2e^−^, which has been proposed to occur at a high proton motive force (pmf)^33^, even though it remains to be demonstrated experimentally. However, experimental data clearly shows that it may reach a maximum of 4 H^+^/2e^−^ in the absence of pmf ^37, 38^. Therefore, a fourth proton transfer pathway may be expected. Our simulation data suggest that a fourth proton channel emerges at the interface of Nqo10 and Nqo11 (Fig. 4 and Supplementary S8). This involves highly conserved Glu32 of Nqo11, which has been found to be indispensable for the activity of the enzyme^39, 40^. Interestingly, this proton uptake route is similar to what has been proposed based on the crystal structure of *Escherichia coli* enzyme and eukaryotic enzyme^17, 19^, but different from the path observed in *T. thermophilus* complex I^18^. A further analysis of the simulation trajectory of setup I reveals that the pathway transiently opens for proton uptake, from the N-side of the membrane, caused by the down flip of conserved Glu32 of subunit Nqo11 (Fig. 4). The exit path for this channel comprises polar residues from Nqo10 and Nqo11 subunits, as shown in Fig. 4. Remarkably, many of the amino acid residues that are predicted to form the fourth proton channel are also found by Kaila *et al*. (ref. 31) in their independent simulation study on *E. coli* complex I (see Supplementary Table S1). We note that the down flip of Glu32 from Nqo11 is not observed in the simulation of setup II, instead it is stabilized in a different conformation hydrogen bonding to the Tyr59 of Nqo10, similar to what is seen in the crystal structure of *Thermus* enzyme^18^. Nevertheless, the agreement between the current and earlier simulations results, as well as the functional importance of Glu32^39, 40^, strongly advocate that the region is involved in proton pumping, and may form the fourth proton pathway.

### Hydration of Nqo8 subunit

**Figure 5:**
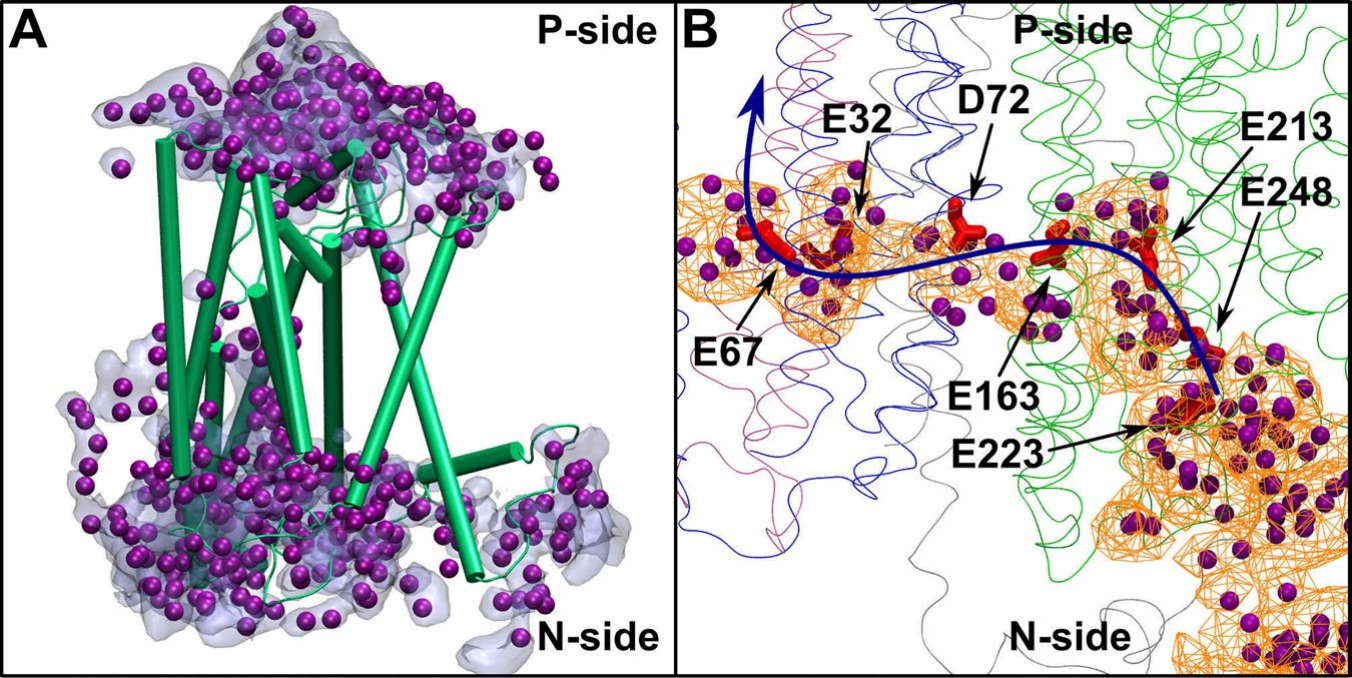
Hydration of the Nqo8 subunit (**A**). Amino acid residues (Glu223, Glu248, Glu213, Glu163 of Nqo8, Glu32, Glu67 of Nqo11, Asp72 of Nqo7 in red) and water molecules (in purple) connect the Q-tunnel with the membrane-bound subunits (**B**). The blue arrow indicates a putative proton pumping route. Water occupancy is plotted with an isovalue of 0.2 (**A**) and 0.1 (**B**). The figure is prepared from simulation setup X in which Glu248, Glu213, Glu163 of Nqo8, and Asp72 and Asp74 of Nqo7 are protonated. In panel A, only water molecules within 4 Å of Nqo8 subunit (green) are shown. In panel B, Nqo7, Nqo8, Nqo10 and Nqo11 are colored grey, green, blue and mauve, respectively, and shown as thin ribbons (see also Supplementary Fig. S9).

The hydration pattern of Nqo8 subunit is most interesting because of very large number of water molecules that enter this subunit during the simulations (Fig. 2). It is observed that part of the subunit hydrates very rapidly (Fig. 5A), whereas the other half remains predominantly dry, mainly due to the presence of large number of hydrophobic residues. This is also observed in longer simulations I and II (Fig. 5 and Supplementary S9). The hydration pattern shows that the section of the Q-tunnel that is embedded in the Nqo8 subunit connects to the middle of the membrane-bound subunits via charged amino acid residues and water molecules (Fig. 5B, see also Fig. 1 inset). In the two longest simulations (setups I and II), a continuous hydrated path is not established (Supplementary Fig. S9). However, when protonation states of acidic residues along this pathway are altered (Table 1), they undergo conformational changes (Supplementary Fig. S3 and Table S3), and recruit water molecules, thereby enabling a continuous connection from the Q-tunnel to the fourth putative proton channel (Fig. 5B). This unique connection extends all the way up to Glu67 of Nqo11 that is ca 35 Å from the Q-tunnel region in the Nqo8 subunit. Interestingly, when electric dipole moment vector of water molecules in this hydrated region is plotted, it is found to align in the direction from the Q-tunnel to the antiporter type subunits (see panel D in Supplementary Fig. S5). We suggest that this connectivity between the middle of the Q-tunnel and the membrane-bound subunits, as observed from simulation data, is critical for the proton pumping function of the enzyme (see below).

**Table 1:** Model systems and simulation lengths.

Model system	Q^1*^	Q^2*^	D139 *Nqo4*	Y87 *Nqo4*	H38 *Nqo4*	E225 *Nqo8*	E223 *Nqo8*	E248 *Nqo8*	E35 *Nqo8*	E213 *Nqo8*	E163 *Nqo8*	D72 *Nqo7*	E74 *Nqo7*	(ns)
I	Q^OX^	—	−1	0	+1	−1	−1	−1	−1	−1	−1	−1	−1	498
II	QH_2_	—	−1	−1	0	−1	−1	−1	−1	−1	−1	−1	−1	585^†^
III	—	QH_2_	−1	−1	0	−1	−1	−1	−1	−1	−1	−1	−1	500^#^
IV	—	QH_2_	−1	−1	0	−1	−1	−1	−1	0	−1	−1	−1	200
V	—	Q^OX^	−1	−1	0	0	0	−1	−1	−1	−1	−1	−1	100
VI	—	Q^OX^	−1	−1	0	−1	0	0	−1	−1	−1	−1	−1	100
VII	—	Q^OX^	−1	−1	0	−1	−1	0	0	−1	−1	−1	−1	200
VIII	—	Q^OX^	−1	−1	0	−1	−1	0	0	0	−1	−1	−1	280
IX	—	Q^OX^	−1	−1	0	−1	−1	−1	−1	0	0	0	0	100
X	—	Q^OX^	−1	−1	0	−1	−1	0	−1	0	0	0	0	200

^*^The Q is modeled as Q^OX^ (oxidized) or QH_2_ (doubly reduced and doubly protonated) at the site close to N2 FeS center (Q^1^) or at another location half-way through the Q tunnel (Q^2^) (see Methods section).

^†^Including five independent ~5 ns simulations.

^#^Including one 266 ns and three independent ~80 ns simulations with QH_2_ modeled at few different locations in the middle region of the Q-tunnel.

### Hydration and dynamics of Nqo4 and Nqo6 subunits

**Figure 6:**
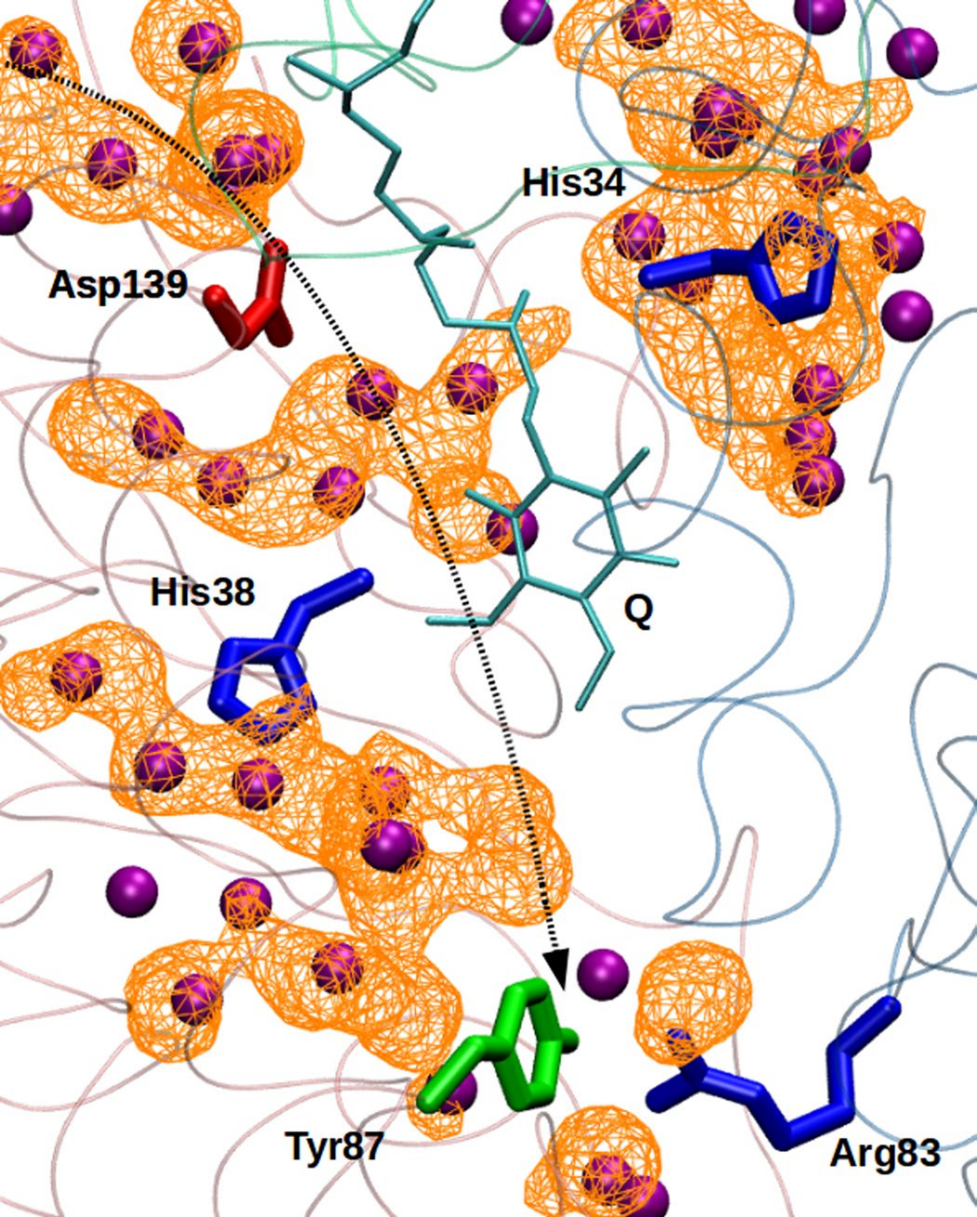
Hydration of the Nqo4 subunit. The water occupancy map is shown as a wireframe plotted with a surface isovalue of 0.2. The key amino acid residues of Nqo4 (His34, His38, Tyr87 and Asp139) and Nqo6 (Arg83), water molecules (purple), and a Q molecule (QH_2_) are also shown. The snapshot is obtained from model # II, and hydrogens are not shown for clarity. The dotted arrow shows a putative (‘premature’) proton transfer pathway towards deprotonated Tyr87 from the bulk (N-side). See also Supplementary Fig. S10.

The two subunits Nqo4 and Nqo6 form the scaffold for the Q-binding close to the N2 FeS center^18, 19^. Due to the presence of multiple charged residues in Nqo4, water molecules rapidly hydrate the subunit (Figs 2, 6 and Supplementary S10), also including the region around the Q head group. This raises an important question that what prevents the protonation of the residues that are left deprotonated after the proton-coupled electron transfer to Q from N2^30^. In an earlier work, a partial stabilization of the anionic Tyr87 by neutral His38 of Nqo4 was observed^30^. However, deprotonated Tyr87 is likely to have a much higher proton affinity, so that it may be protonated by the proton released upon QH_2_ oxidation (see also Methods), which would result in a loss of free energy of proton pumping. Also, the protonation of the anionic Tyr (and His38/Asp139 ion pair) from the N- or the P-side of the membrane prior to the completion of the pump cycle may destabilize conformational states necessary for proton translocation.

We suggest that Arg83 from Nqo6 electrostatically stabilizes the anionic Tyr87, as observed in our multiple independent simulations (Fig. 6, Supplementary Fig. S11, and Table 1). We note that this arginine is not fully conserved, instead replaced by a threonine in complex I from other organisms such as *Bos taurus*, *Escherichia coli* and *Yarrowia lipolytica*. However, our analysis of recent cryo-EM structure of mammalian complex I^20^ reveal that an arginine residue (Arg85, bovine numbering, PDB id: 5LC5) from 49 kDa subunit effectively compensates for the missing arginine in the PSST subunit, and forms a hydrogen bonding interaction with Tyr108 (bovine numbering) (Supplementary Fig. S11). Interestingly, the latter arginine residue in bovine complex I has been suggested to be functionally critical based on mass-spectroscopy data^41^. We hypothesize that the position of Arg is swapped in the two enzymes classes (e.g. *Thermus* and *Bovine*), but the functional role is shared. This viewpoint is also supported by the coevolution analysis performed on Nqo4/6 sequences using two different tools, GREMLIN^42^ and EVcomplex^43^. The data from GREMLIN (normalized coupling strength >1) and EVcomplex (EVcomplex score ~0.53) suggests that Thr64 in Nqo4 and Arg83 in Nqo6 are evolutionarily coupled such that their positions are swapped in other organisms, without altering the function of Arg83 (see above). Such positional swapping of functionally critical residues is well-known in the heme-copper oxidase superfamily^44, 45^. We suggest that the stabilization of the deprotonated form of Tyr87 is necessary to prevent protonic equilibration between the N-side (or the P-side) of the membrane and the latter residue, which would otherwise result in loss of proton pumping.

### Protonation dependent side chain dynamics

**Figure 7:**
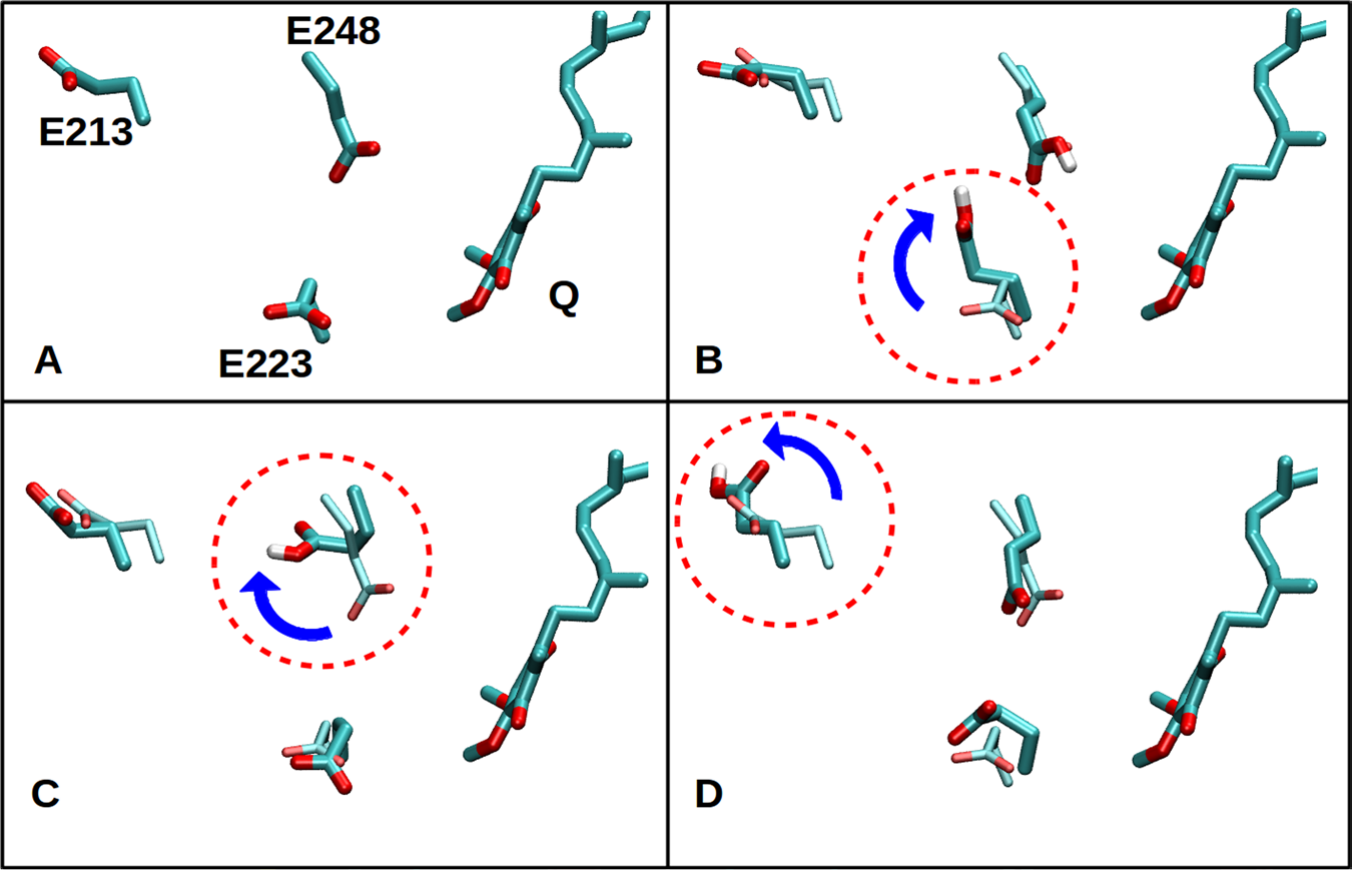
Flipping of amino acid side chains as observed in MD simulations. (**A**) A simulation snapshot (setup III in Table 1) shows QH_2_ (hydrogens not shown) close to the deprotonated acidic residues Glu248 and Glu223. Protonation of acidic residues (upon QH_2_ oxidation) results in flipping of the side chains of protonated Glu223 (**B**), protonated Glu248 (**C**), and protonated Glu213 (**D**) in simulations setups VI, VII and IV, respectively (see also Table 1). The QH_2_ molecule position is same in all panels, and is based on setup III simulation.

The hydration pattern observed for Nqo8 subunit in our atomistic simulations show that the water molecules together with charged residues establish a connection between the middle of the Q-tunnel and the negatively charged residues of Nqo7–8 subunits (see Fig. 5B, Supplementary Fig. S5D and inset of Fig. 1). We propose that this pathway may be used to transfer protons (e.g. released upon proposed QH_2_ oxidation, see ref. 2) to the P-side of the membrane. In order to shed light on the latter aspect, we performed MD simulations by changing the protonation states of the residues that line up along the path from the Q-tunnel to the membrane-bound subunits (see Fig. 5B, Table 1 and Methods section). We observe that change in the protonation state of key acidic residues bring up on a large change in their sidechain conformations (Fig. 7 and Supplementary Fig. S3). This is also displayed as the root mean square fluctuation (RMSF) of the sidechains of selected amino acids (Supplementary Table S3). It is observed that the RMSF of protonated Glu223 and Glu225 in setup V, protonated Glu223 and Glu248 in setup VI, protonated Glu35 and Glu248 in setup VII, and protonated Glu35, Glu223 and Glu248 in setup VIII is consistently higher than the RMSF of same residues in setup III (model system from which all the former systems were constructed, see Methods section). Similarly, a comparison of the RMSF values of protonated Glu163 and Asp72 in systems IX and X reveal larger fluctuations relative to the setup VIII. In particular, Glu223, Glu248, and Glu213, undergo flip of their sidechains, thereby providing a water-based connectivity between the latter site at the Nqo4/6/8 interface and the membrane-subunits, that is encompassing a distance of ca. 35 Å between the Q-tunnel and the first putative proton exit route at Nqo11 (Figs 5B and 7). We also note that flipping of the sidechain of protonated amino acid residues residues, Glu223 (setup VI), Glu225 (setup V), Glu163 (setup IX), Glu248 and Glu35 (setup VII) of Nqo8 subunit (Supplementary Fig. S3, panels C and D) coincides with the changes in their p*K*_a_ values (Supplementary Fig. S12).

### Behavior of the modeled quinone (Q) molecule

Earlier based on atomistic MD simulations, redox-dependent conformational changes in Nqo4 subunit were reported^30^. The current simulation data support these observations, but in addition to that highlights the important conformational changes that occur in the Nqo6 subunit. In the simulation setup II, which represents the state formed after redox-coupled protonation of bound Q near N2 center, Arg83 from Nqo6 subunit is found to stabilize the anionic Tyr87 in multiple independent simulations (Fig. 6). This occurs together with the dissociation of His-Asp ion-pair, and migration of His38 towards anionic Tyr87, a notion also supported by the structural data (PDB id: 4WZ7, see also ref 30).

The average RMSF of the Q head group (average of six aromatic carbon atoms of the ring) is found to be 2–3 Å when modeled at the site close to N2 center. On the other hand, when Q is modeled in the middle of the Q-tunnel, the RMSF is found to be slightly lower (0.7 to 2.0 Å). At any rate there are no large scale conformational changes in the Q behavior in these simulation time scales, except that the hydrophobic tail (Q_6_) fluctuates, either when it is partly buried or completely buried in protein interior. In contrast to the mild conformational changes in Q, the protonation of residues (Glu223, Glu225, Glu248 from Nqo8) shows large scale changes in their sidechain movements, also with respect to the relatively fixed Q head group position (Supplementary Fig. S13).

## Discussion

Fully atomistic classical MD simulations performed on the entire structure of respiratory complex I from *T. thermophilus* reveal rapid hydration of the membrane-bound subunits, and the Q-binding site region. Based on simulation data we find that transient water-based connections form in the antiporter-like subunits (Nqo12–14), which may play an important role in proton uptake. Interestingly, a complete S-shaped connectivity encompassing the entire antiporter-like subunits is never observed, which may be necessary to minimize proton leaks from the P-side to the N-side of the membrane^46^. It is to note that many of the amino acid residues that are proposed to form part of the proton conducting pathway are known to be critical based on site-directed mutagenesis data^24–28^. Furthermore, consistent with the 4 H^+^/2e^−^ pumping stoichiometry of complex I and structural data, the simulation results show that the fourth proton channel emerges at the interface of Nqo10 and Nqo11 subunits. Overall, our results on proton transfer pathways in the antiporter type subunits, and another one at the Nqo10/11 interface, agree well with the earlier simulation study on complex I from *E. coli*.^31^

The hydration pattern of Nqo8 subunit is particularly unique in that it reveals water-based connectivity between the Q-tunnel and the fourth proton channel at the interface of Nqo10 and Nqo11, thereby encompassing a distance of more than 30 Å. The conformational dynamics of the sidechains of acidic residues in Nqo8 subunit observed in our simulations is also partly supported by the structural data; some of these acidic residues are found to undergo sidechain (or backbone) conformational transitions, as observed in the crystal or cryo-EM structures of complex I (Supplementary Fig. S14).

The functional significance of the hydration observed in Nqo8 subunit also calls for a brief note. It is well-known that the mitochondrial analogue of Nqo8 subunit (see also Supplementary Table S1) is a ‘hot-spot’ for point mutations^47^. The hydration pattern and sidechain dynamics observed in our simulations strongly suggest that this region of complex I plays an important role in coupling between the Q redox chemistry and proton pumping, which when disrupted by point-mutations, may result in suboptimal complex I function, causing mitochondrial disorders.

The region close to the Q-binding site, formed by Nqo4, Nqo6 and Nqo7 subunits, hydrates rapidly. In such a scenario, it is not easy to envisage how the charge accumulated in the Q-binding cavity upon Q reduction would remain uncompensated by the bulk protons that is until the completion of a single turnover (milliseconds). Based on MD simulations of the state formed immediately after reduction-coupled protonation of Q (model system # II), we find that the anionic Tyr is stabilized by Arg83 from Nqo6. We suggest that this may be the mechanism to prevent the premature protonation of tyrosinate (deprotonated Tyr), which would result in loss of free energy of proton pumping. We note that some of the key residues (Tyr87 from Nqo4 and Arg83 from Nqo6) are only 6 Å from the N2 FeS cluster, and their dynamics and acid-base properties may be influenced by the redox/acid-base properties of the FeS cluster. Such subtle aspects, including polarization effects, are not taken into account in the current study, but may play an important role in enzyme mechanism^48^.

One of the key results from our simulations is the hydration of the region between the Q-tunnel and the proton pathway closest to it. Though this connectivity is partially visible in the crystal structures, our simulations reveal that the large gaps between the charged amino acid side chains are filled with dynamic water molecules (Fig. 5B). The observed water occupancy together with the sidechain dynamics of key residues completes the network, thereby highlighting the importance of protein hydration and dynamics in enzyme function. An earlier mechanistic proposal supported the role of Q as a dynamic electron carrier permanently trapped in the Q-tunnel^2^. The data presented here points to an additional role of trapped Q in translocating protons from the N-side to the P-side of the membrane, such that the water-protein based connectivity from Nqo8 to Nqo10/11 may be used to transfer protons released upon QH_2_ oxidation, thereby supporting the viewpoint that part of the coupling may be ‘direct’ in nature.

The basic design principles of redox-coupled proton pumping in complex I are not known^2^. Our simulation data provide a significant advancement in this regard, and show how protein and water dynamics may be important in long-ranged electron-proton coupling in complex I. Our mechanistic proposals are tentative but testable, and call for further experimental (e.g. site-directed mutagenesis of Arg83 from Nqo6) and theoretical work to prove or disprove it.

## Methods

### Model system construction and simulation setup

We performed all-atom classical MD simulations on the respiratory complex I from *Thermus thermophilus* (PDB ID: 4HEA)^18^. Earlier MD simulation studies^31, 35^ were performed on the membrane domain of the *E. coli* enzyme. Therefore, as a next relevant step, and in continuation to earlier work^30^, we have performed atomistic simulations on the entire structure of complex I. The model systems comprised entire complex I embedded in a 1-palmitoyl-2-oleoylphosphatidylcholine (POPC) lipid bilayer, TIP3 water molecules, and Na^+^ and Cl^−^ ions mimicking the 100 mM salt concentration. The Q-binding site was modeled with a Q_6_ molecule in two different states (fully oxidized quinone and doubly-protonated quinol, QH_2_), as described earlier^30^. The resulting system consisted of approximately ~850000 atoms. The simulation program NAMD^49^ together with the CHARMM36 force field for protein, lipids, water and ions^50, 51^ were used to perform the simulations. The force field parameters for modelled Q^52^, reduced FeS clusters^53^, FMN^54^ and deprotonated Tyr^55^ were used as described earlier^30^. The N2 FeS cluster, which is the immediate electron donor to Q, has relatively high midpoint potential among all FeS clusters, ensuring its maximal electron occupancy^8^. For this reason, as well as for simplicity, we have modeled all FeS clusters in reduced state. It is also assumed that the redox state of FeS clusters does not influence the dynamics of water molecules in the membrane-bound subunits.

For model systems I and II, a 1000 step energy minimization was performed while keeping the protein fixed. The position of Q in the Q-tunnel was refined with short minimizations and simulations (rest of the system fixed). This was followed by an energy minimization of the entire system, and a 1 ns NVT simulation in which lipid tails were allowed to relax (protein backbone and other cofactors fixed). For production runs some atoms of the FeS clusters and FMN were kept restrained to their crystallographic positions using harmonic force constants of 50 kcal mol^−1^ Å^−2^. The pressure (1 atm) and temperature (310 K) were controlled with the Nosé-Hoover Langevin barostat and Langevin thermostat^56–58^, respectively, as implemented in NAMD. A timestep of 1 fs was employed. The long-range electrostatics was treated with the use of particle mesh Ewald technique (PME)^59^, as available in NAMD.

### Rationale for additional simulation setups

We observed extensive hydration in the Nqo8 subunit in the two longest simulations I and II (see also Results section, and Fig. 5A), that is closer to the residues Glu163 and Glu213. A water-based connectivity is also observed between the latter residues, and the acidic residues in the middle of the Q-tunnel (Glu248, Glu223 and Glu225 of Nqo8). In an earlier work^2^, it was proposed that a quinol (QH_2_) molecule (formed after two-electron reduction from N2, and protonation from His38 and Tyr87) slides to another position somewhat in the middle of the Q-tunnel, where it gets oxidized to another loosely-bound Q at the membrane-protein interface. Even though there is no structural information for the proposed position in the middle of the Q-tunnel, we performed additional MD simulations (system III in Table 1) by modeling a QH_2_ molecule next to the acidic residues Glu223, Glu225 and Glu248 that lie in the middle of the Q-tunnel. The latter acidic residues belong to the segment (Asp220–Lys282 of Nqo8), which has been identified as the inhibitor-binding site based on photo-affinity labeling of bovine mitochondrial complex I^60^, see also ref. 2.

We also performed additional simulations by modeling an oxidized Q at the same site, but by redistributing the two protons (released upon QH_2_ oxidation) on nearby acidic residues (see Table 1). We emphasize that these residues in the middle of Nqo8 and Nqo7 subunits are known to be functionally important based on the site-directed mutagenesis data^47, 61–63^. All model systems (IV–VIII) were constructed by using the snapshot from the simulation of system III. Models III and IV are exactly the same except for the protonated Glu213 in setup IV, which was suggested to be the case in an earlier work^30^. The two protons released upon proposed oxidation of QH_2_ molecule (by a loosely-bound Q)^2^ are redistributed on to two nearby residues Glu223 and Glu225 (system V), on Glu223 and Glu248 (system VI) and on Glu35 and Glu248 (system VII). The system VIII is same as system VII, but with Glu213 protonated, see ref. 30.

Since the region close to Glu248 and Glu223 of Nqo8 connects to the acidic residues of Nqo7, via protonated Glu163 and Glu213 of Nqo8 and water molecules, we altered the protonation states of Asp72 of Nqo7, which has been found to be important based on site-directed mutagenesis data^64^, and constructed the systems IX and X (from setup VIII coordinates). The residue adjacent to Asp72 of Nqo7 is Glu74, and is known to be functionally not critical^64^. Also, based on the crystal structure analysis, we decided to leave Glu74 protonated in both the simulations IX and X. Assuming that Glu213 and Glu74 are protonated in the ground state of the enzyme, setup IX would represent a state in which the two protons released upon QH_2_ oxidation reside on Glu163 and Asp72 of Nqo8 and Nqo7, respectively, whereas setup X would represent a scenario in which two released protons reside on Glu248 of Nqo8 and Asp72 of Nqo7, and Glu213, Glu163 and Glu74 are neutral in the ground state (see also ref. 30).

Simulation data were analysed with the visualisation program VMD^65^. The total simulation time is ca. 2.8 μs. Simulation snapshots are uploaded on the open-access research data repository *Zenodo*, and can be downloaded from the link (http://doi.org/10.5281/zenodo.267916). Residue numbers mentioned in Supplementary Table S1 were used for the data shown in Fig. 2. First, a set of residues were chosen based on the visual analysis of the water pathways. After this, only those polar residues were selected, which were found to be highly conserved, and functionally important based on site-directed mutagenesis data (see Introduction).

Electrostatic calculations were performed using PROPKA software (version 3.1)^66, 67^ to calculate the p*K*_a_ of titratable residues. Simulation snapshots (every ns) comprising heavy atoms of subunits Nqo4, Nqo7, Nqo8, Nqo10, Nqo11 and Nqo12–14 were used to perform the p*K*_a_ calculations. The p*K*_a_ data is shown in Supplementary Table S2, and is in overall agreement with the standard protonation states considered for residues in membrane-bound subunits (setups I and II).

### Data Availability

The datasets (simulation snapshots) generated during the current study are available in *Zenodo* repository [http://doi.org/10.5281/zenodo.267916].

## Electronic supplementary material

Supplementary Material
