# Supplementary material for: Role of water and protein dynamics in proton pumping by respiratory complex I
Source: Sci Rep. 2017 Aug 10;7:7747. doi: 10.1038/s41598-017-07930-1 (PMC5552823; doi:10.1038/s41598-017-07930-1)
Supplement: Supplementary file 1 — Supplementary Material [file 41598_2017_7930_MOESM1_ESM.pdf]

Supplementary Information for

Role of water and protein dynamics in proton pumping by respiratory complex I

Outi Haapanen[a,b], Vivek Sharma[a,b,c,*]

[a] Department of Physics, University of Helsinki, P. O. Box 64, FI-00014, Helsinki, Finland.

[b] Department of Physics, Tampere University of Technology, P. O. Box 692, FI-33101, Tampere, Finland.

[c] Institute of Biotechnology, University of Helsinki, Finland

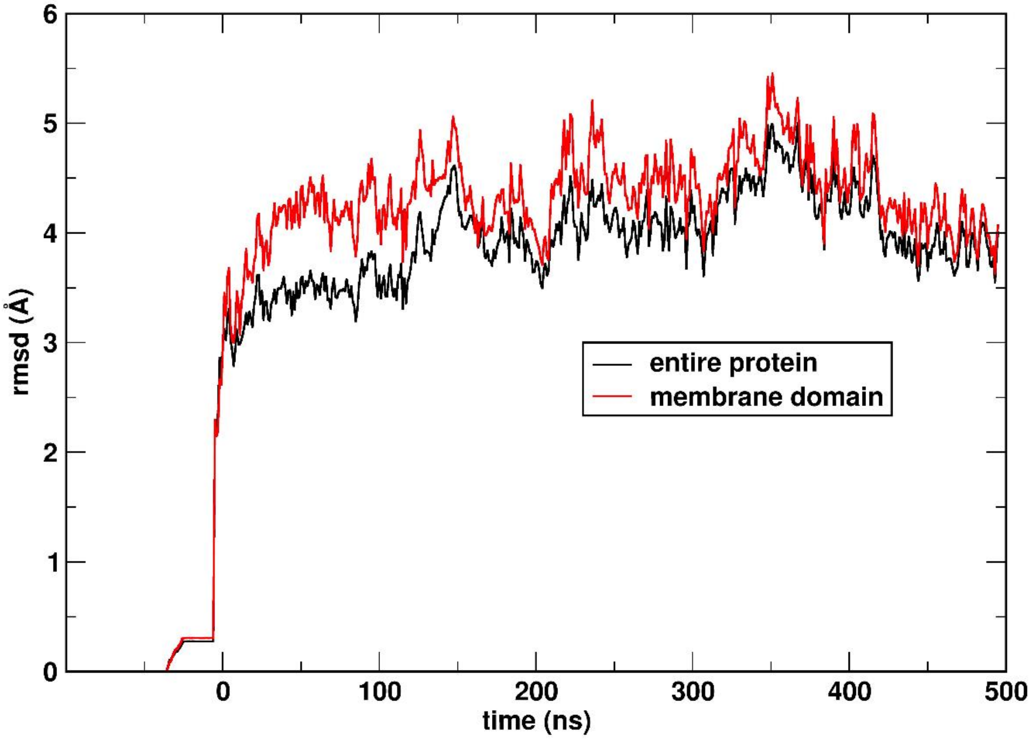

Supplementary Figure S1. RMSD of entire protein and the membrane domain from simulation I. The region $x < 0$ corresponds to minimization and constrained simulation (see Methods section). The RMSD is calculated by aligning the entire trajectory on to the zeroth frame using Cα atoms of all subunits.

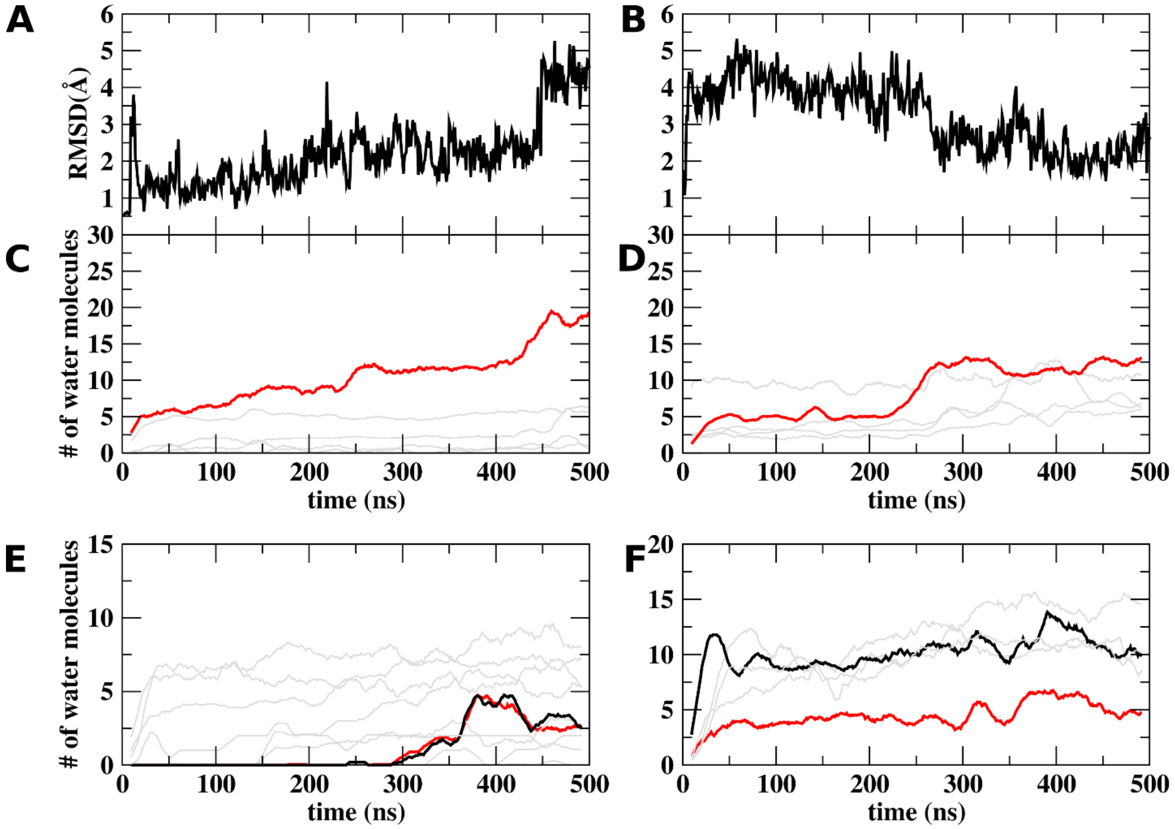

Supplementary Figure S2. Variations in the hydration profiles. The flipping of sidechains (change in RMSD) of His211 (A) and Lys216 (B), of Nqo13 and Nqo14, respectively, occurs simultaneously with the increase in hydration in the regions including the residue or next to it (red plots in panels C and D, see also Fig. S3). The hydration in other regions remains constant (grey plots). The regions selected for this analysis are discussed in Table S1. E and F) Hydration-per-residue in subunits Nqo7/10/11 and Nqo8. E) The transient hydration (followed by dehydration) at around 400 ns occurs primarily next to the residues Asn35 (black) and Asn36 (red) of Nqo10 and Nqo11, respectively (see also black curve in Fig. 2 for Nqo7/10/11 subunits). F) Similarly, an increase (followed by decrease) in hydration around residues Glu213 (black) and Glu35 (red) of Nqo8 occurs at around 400 ns (see also black curve in Fig. 2 for Nqo8 subunit). The grey profiles in plots E and F correspond to other residues of subunits Nqo7/10/11 and Nqo8 (see Table S1), which do not show any major change in hydration at around 400 ns (see text). The hydration profiles in panels C – F are smoothened by a running average of 20 simulation snapshots to improve clarity.

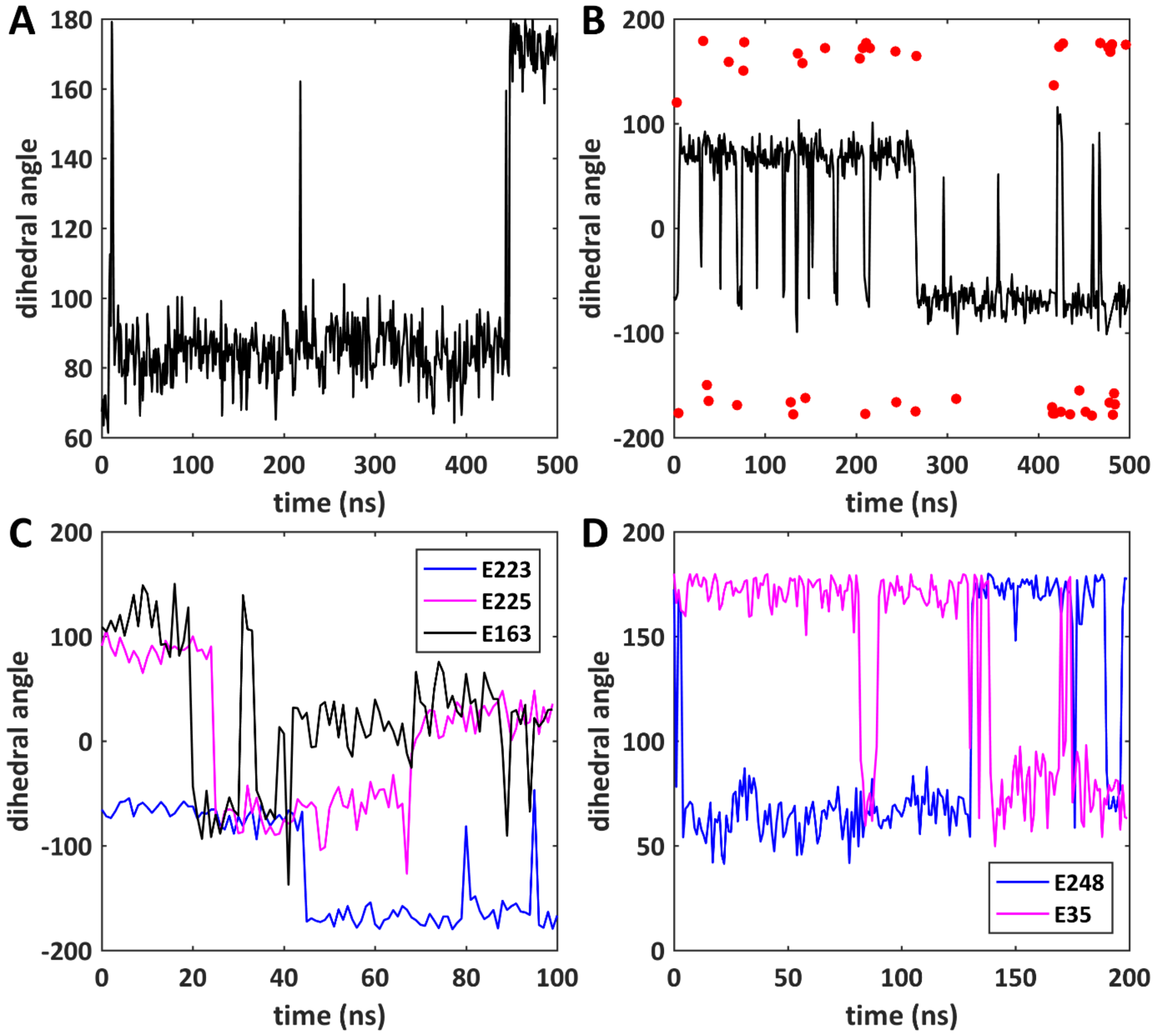

Supplementary Figure S3. Flipping of amino acid side chains with respect to simulation time. The sidechain dihedrals shown are; (A) N-Cα-Cβ-Cγ or χ_1 of His211 (Nqo13) in setup II, (B) Cγ-Cδ-Cε-Nζ or χ_4 of Lys216 (Nqo14) in setup I, (C) N-Cα-Cβ-Cγ or χ_1 of Glu223 (Nqo8) in setup VI, O=C-Cα-Cβ for Glu225 (Nqo8) in setup V, and Cβ-Cγ-Cδ-Oε or χ_3 of Glu163 (Nqo8) in setup IX, and (D) N-Cα-Cβ-Cγ or χ_1 of Glu248 (Nqo8) and Cα-Cβ-Cγ-Cδ or χ_2 of Glu35 (Nqo8) in setup VII. The flip in χ_1 and χ_4 of His211 and Lys216 of Nqo13 and Nqo14, respectively, occurs simultaneously with the increase in their sidechain RMSDs as well as increase in hydration in their vicinity (see Fig. S2 and main text). In panel B, the values of χ_4 closer to ± 180° are shown as red dots, separate from the main plot (black), for the sake of clarity. The change in sidechain dihedrals of Glu223, Glu225, Glu163, Glu248 and Glu35 of Nqo8 bring shifts in their pK_as (see Figure S12).

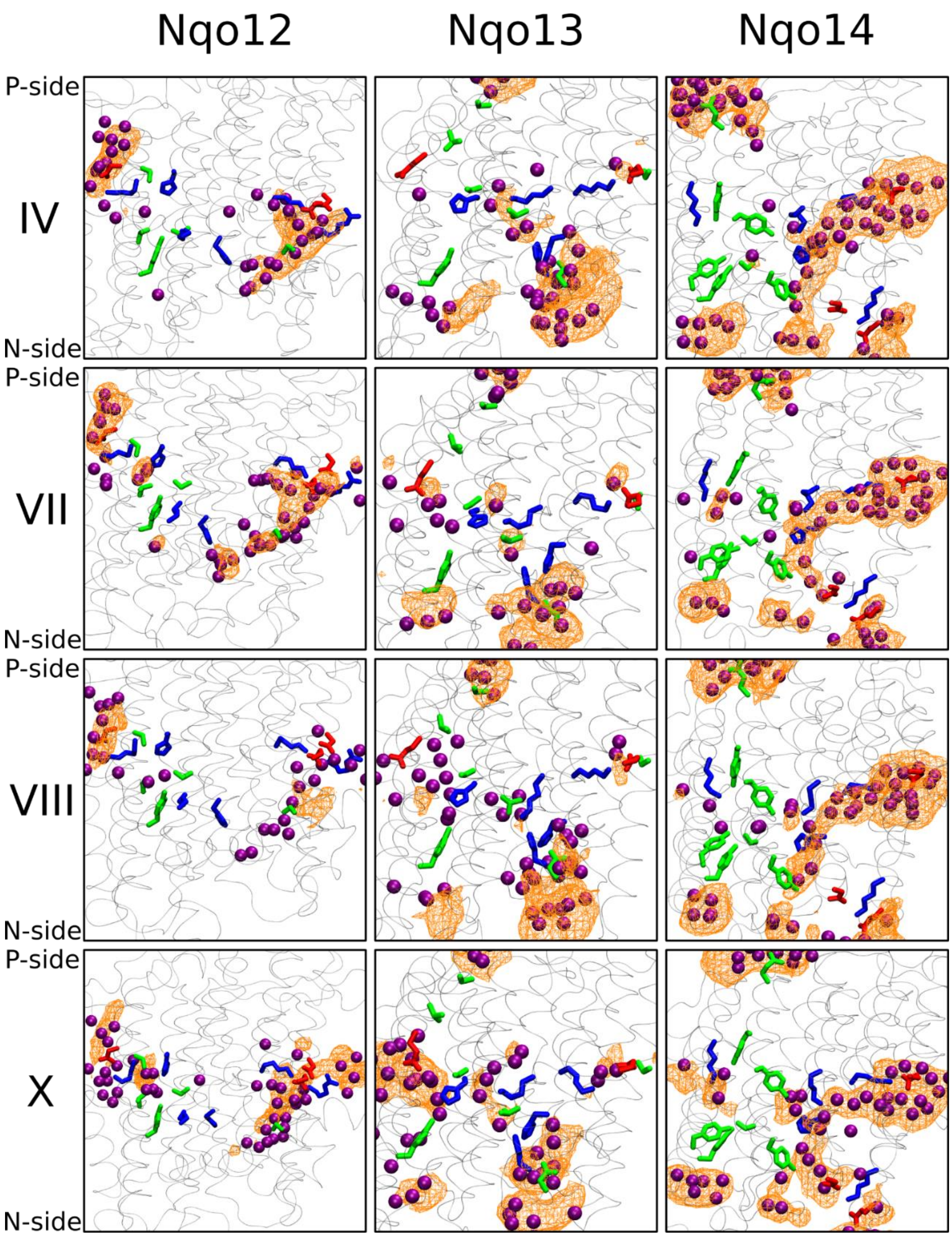

Supplementary Figure S4. Water occupancy (orange mesh with surface isovalue of 0.1) in Nqo12, Nqo13 and Nqo14 subunits from simulations IV, VII, VIII and X. The water molecules (purple spheres), acidic (red), basic (blue) and polar (green) amino acids together with the subunit (thin grey ribbons) are displayed. The P- and the N-side of the membrane are also marked.

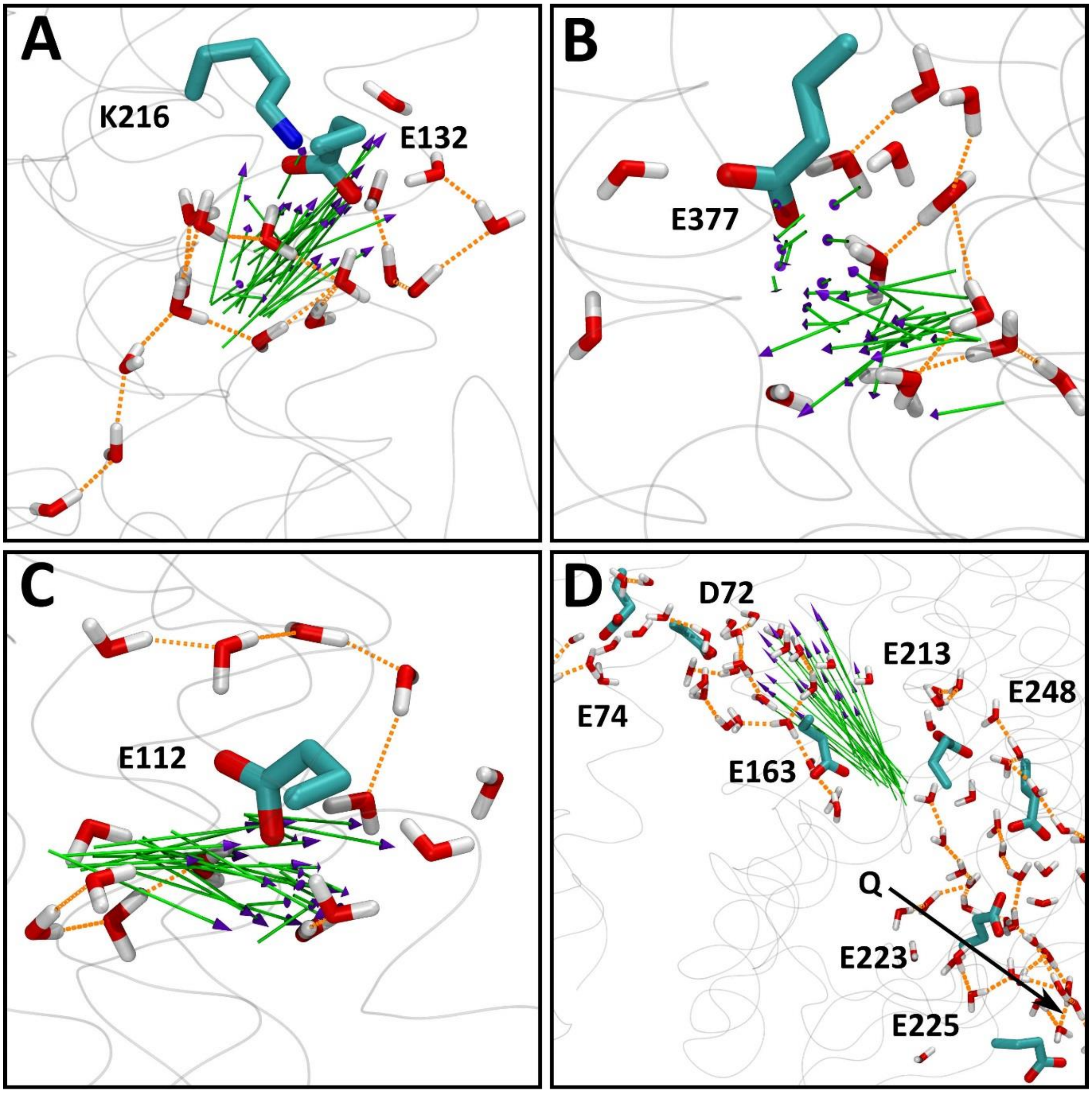

Supplementary Figure S5. Instantaneous electric dipole moment of water molecules in several hydrated regions of protein subunits Nqo12 (A), Nqo13 (B), Nqo14 (C) and Nqo7/8 (D). The total electric dipole moment vector of water molecules is shown as a green/purple arrow, plotted every 15 ns from the last 400 ns of simulation data of setup I (A, C and D) and setup II (B). Hydrogen bonds between water molecules are shown as orange dotted lines based on the criteria donor (D) – acceptor (A) distance ≤ 3.5 Å and D-H-A angle > 150 º. A putative Q-binding site in the middle of the Q-tunnel is marked by an arrow in panel D. Key charged amino acid residues are labeled. Carbon (cyan), oxygen (red), nitrogen (blue), and hydrogen (white) atoms are shown, along with the protein backbone in transparent grey ribbon.

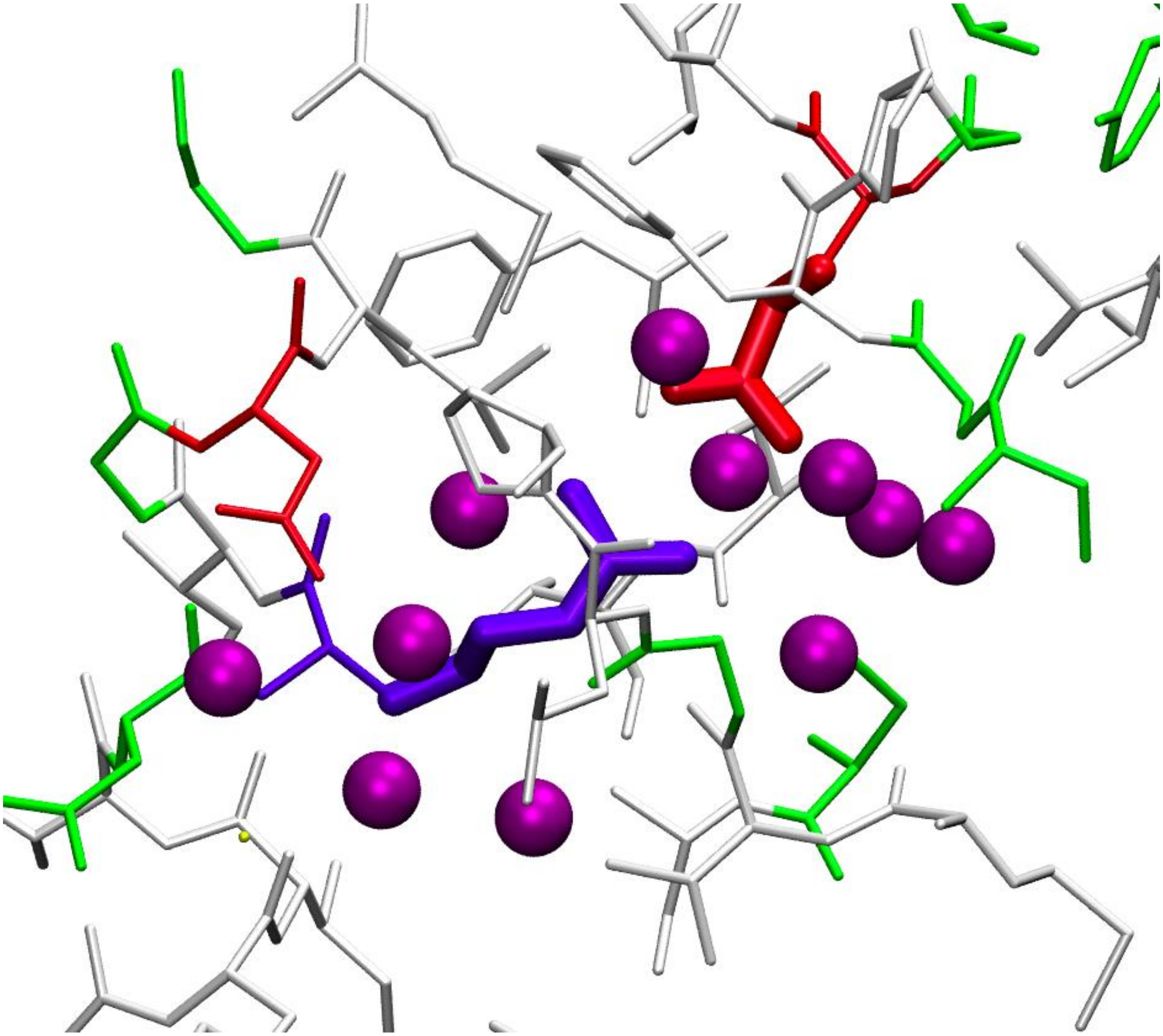

Supplementary Figure S6. The ion-pair between Arg163 (Nqo12) and Glu377 (Nqo13) from simulation setup I. Acidic (red), basic (blue), non-polar (white) and polar (green) residues are displayed, along with the water molecules (purple). Hydrogens are not shown for clarity.

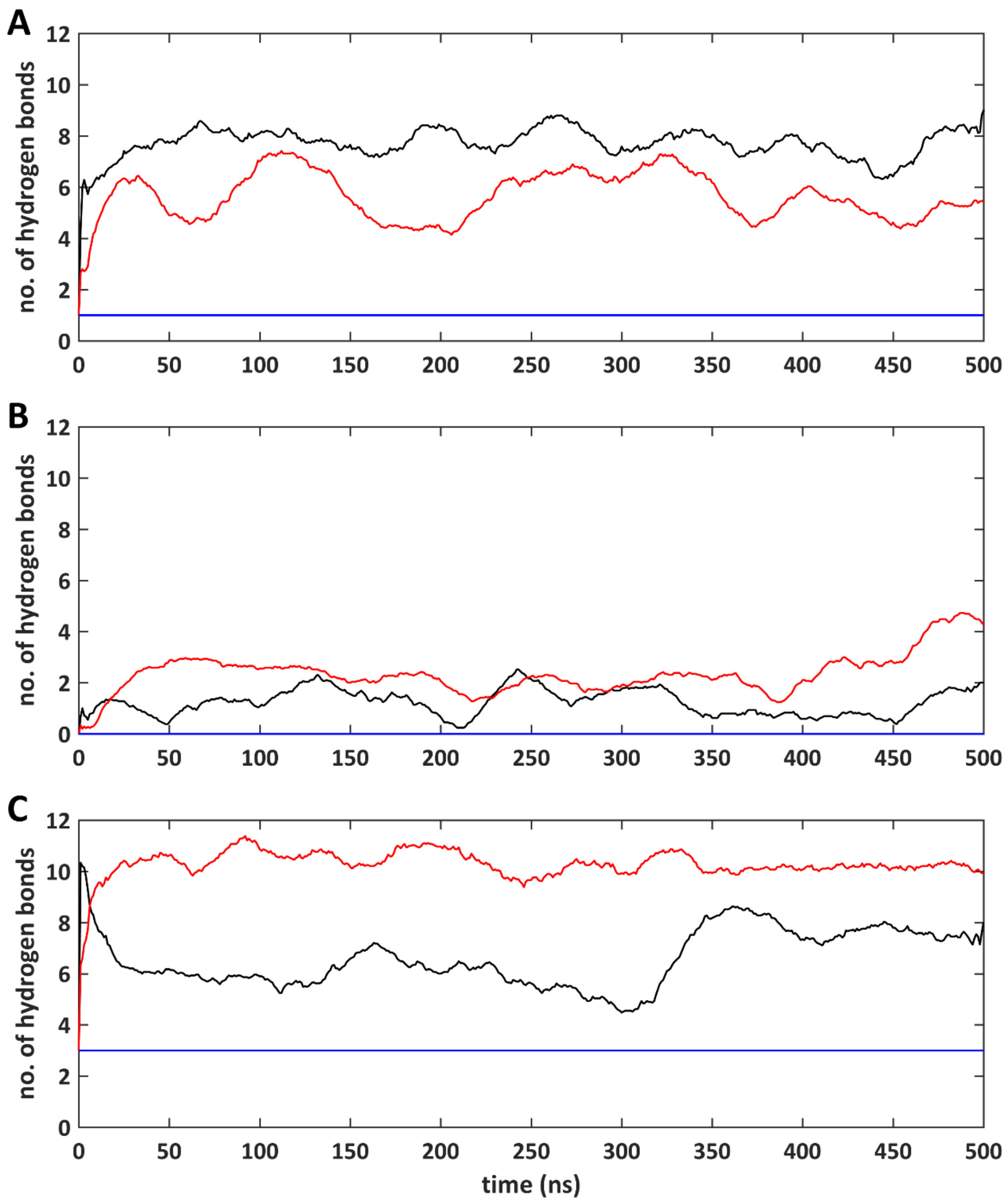

Supplementary Figure S7. Number of hydrogen bonds between Nqo12 and Nqo13 (A), Nqo13 and Nqo14 (B), and Nqo14 and Nqo7/10/11 (C), as a function of simulation time from setups I (black) and II (red). The blue lines indicate the number of hydrogen bonds present in the crystal structure conformation. The long horizontal helix of Nqo12 was excluded from the calculation. Except Nqo12/13 interface (A), all hydrogen bonds were found to form between the loops of the subunits.

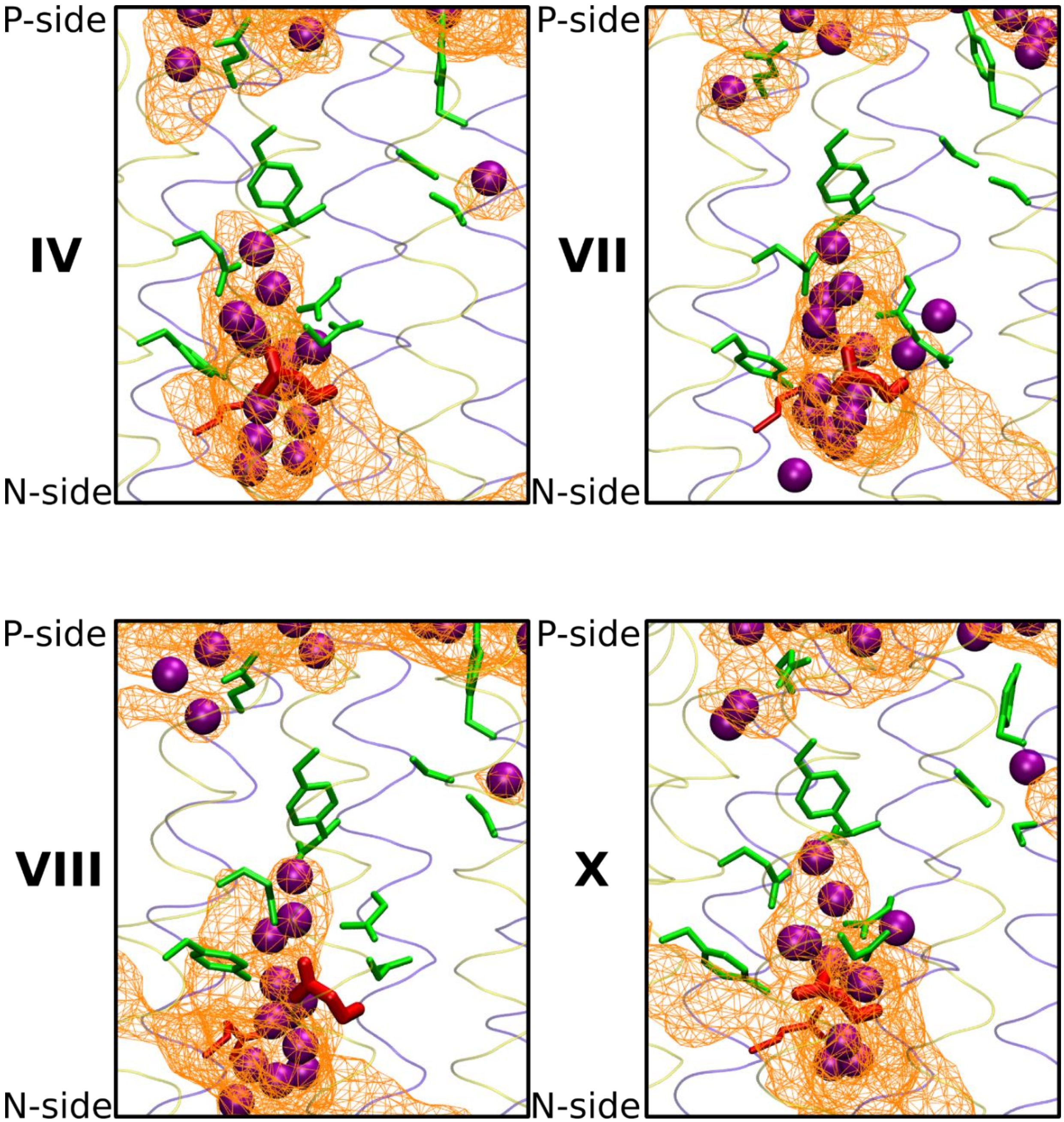

Supplementary Figure S8. Water occupancy map (orange) at an isovalue of ~ 0.1, and instantaneous water positions (oxygen atoms in purple) are shown in the fourth putative proton channel. The acidic (red) and polar (green) residues are shown in licorice representation. Subunits Nqo10 and Nqo11 are shown in yellow and blue ribbons, respectively. The water occupancy is calculated from the trajectories of simulations IV, VII, VIII and X (Table 1) by averaging positions of water molecules within 5 Å of the polar residues of helices in Nqo10 and Nqo11 subunits.

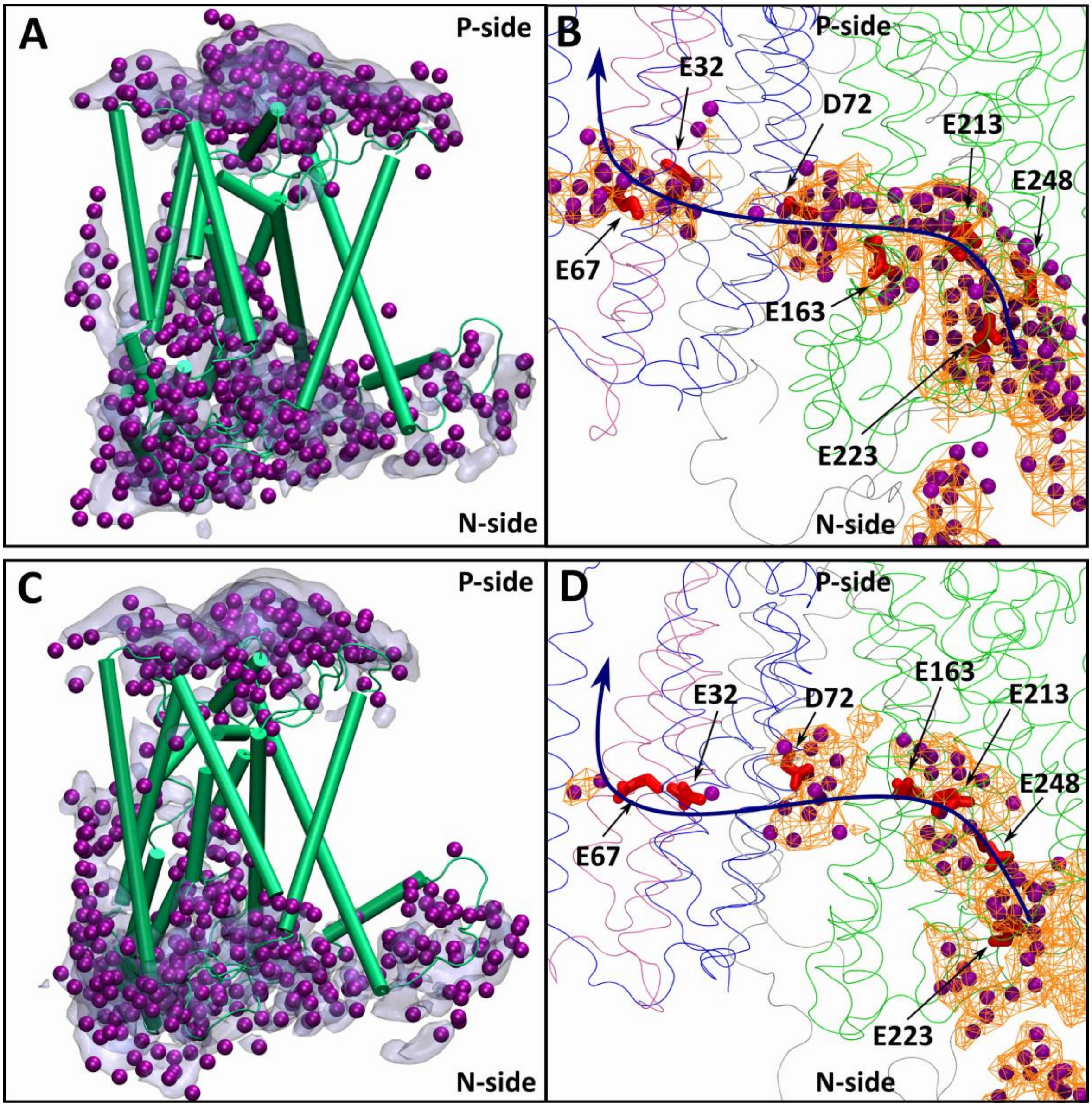

Supplementary Figure S9. Overall hydration of the Nqo8 subunit (green) shown in A (setup I) and C (setup II). The water molecules within 4 Å of Nqo8 subunit are shown as purple spheres, and grey occupancy map is plotted at an isovalue of 0.2. The putative proton transfer path is shown with a blue arrow in panels B (setup I) and D (setup II). Orange mesh represents water occupancy averaged over the entire simulation, and is plotted at an isovalue of 0.1. Key acidic residues (deprotonated state) involved in this pathway are shown in red. The connectivity between the Q-tunnel and the antiporter-type subunits is not complete in simulations I and II, but see Fig. 5 and main text. In panels B and D, subunits Nqo7, Nqo8, Nqo10 and Nqo11 are colored grey, green, blue and mauve, respectively.

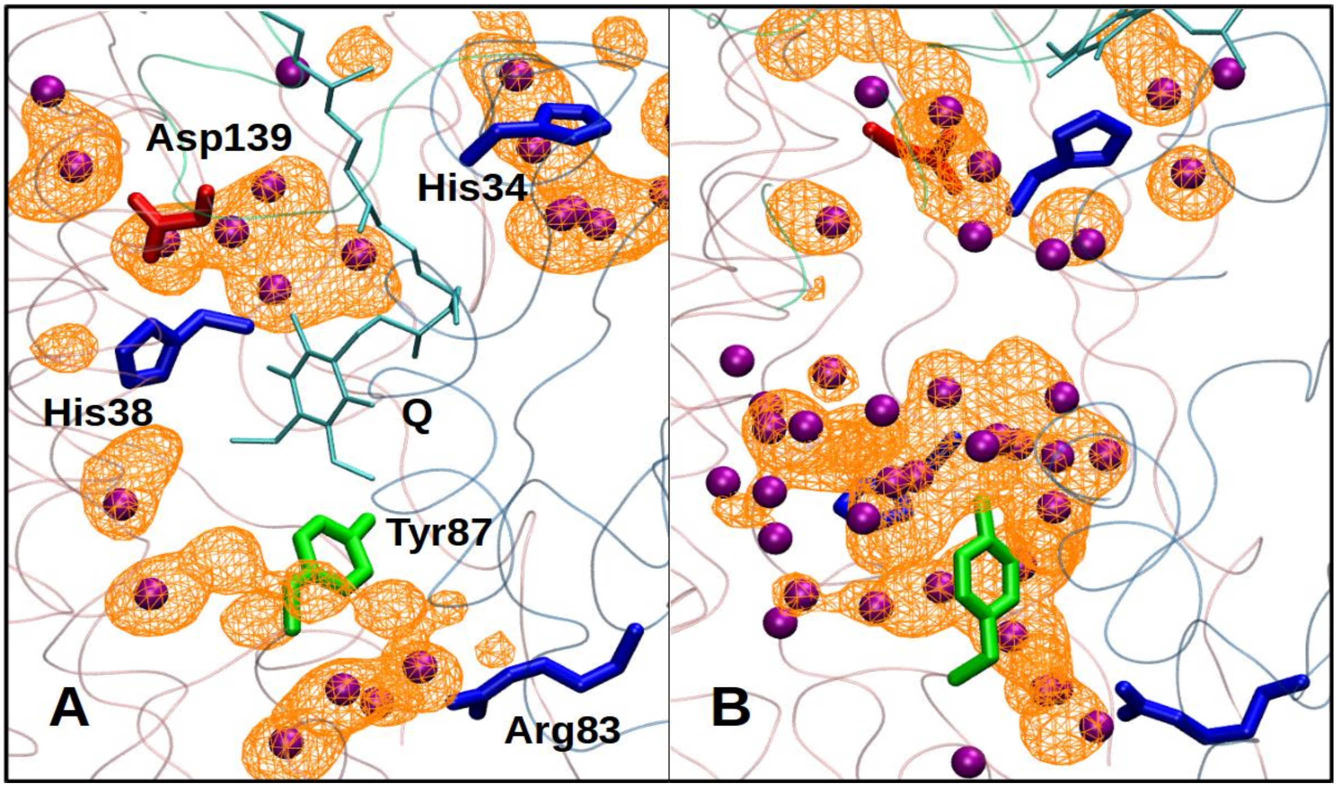

Supplementary Figure S10. Hydration of the Nqo4 subunit in simulation setups I (A) and III (B). The water occupancy map, shown as an orange mesh, is plotted at an isovalue of 0.2. Tyr87, His38, Asp139 and His34 from Nqo4, and Arg83 from Nqo6 subunit are displayed. Q molecule shown in (A) is an oxidized quinone (see also Table 1 in main text).

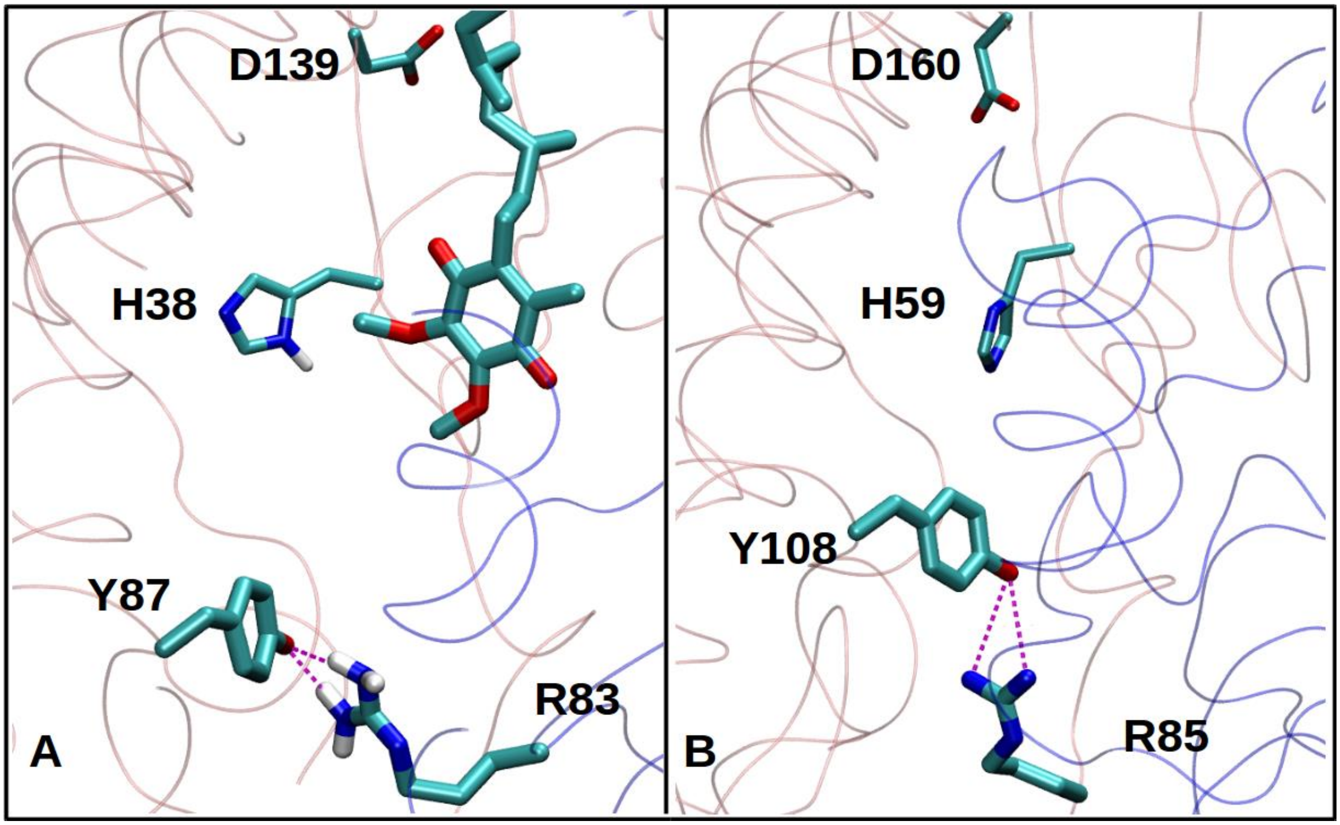

Supplementary Figure S11. Stabilization of deprotonated Tyr. A) Snapshot from simulation (#II) showing stabilization of anionic Tyr87 of Nqo4 (pink) by Arg83 from Nqo6 subunit (blue). A quinol molecule (hydrogens not shown) is also displayed. B) Cryo-EM structure of the respiratory complex I from *Bos taurus* display an analogous interaction between the Tyr108 and Arg85 of 49 kDa subunit. Amino acid numbering corresponds to the enzymes from *T. thermophilus* (A) and *B. taurus* (B). Hydrogen bonding is shown by purple dotted lines.

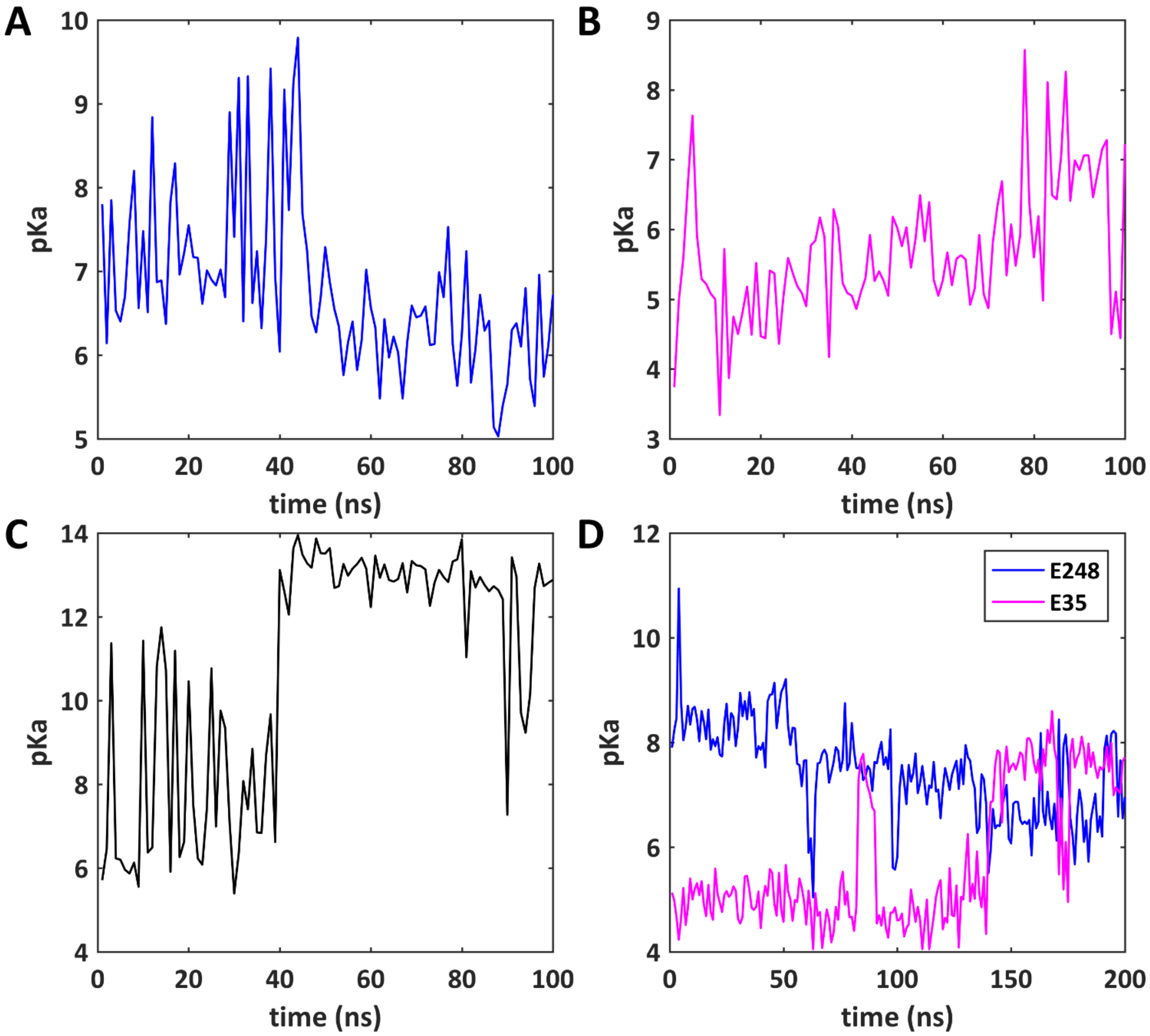

Supplementary Figure S12. The change in pK_a with respect to simulation time is shown for (A) Glu223 (Nqo8) from setup VI, (B) Glu225 (Nqo8) from setup V, (C) Glu163 (Nqo8) from setup IX, and (D) Glu248 and Glu35 (Nqo8) from setup VII. See also Fig. S3.

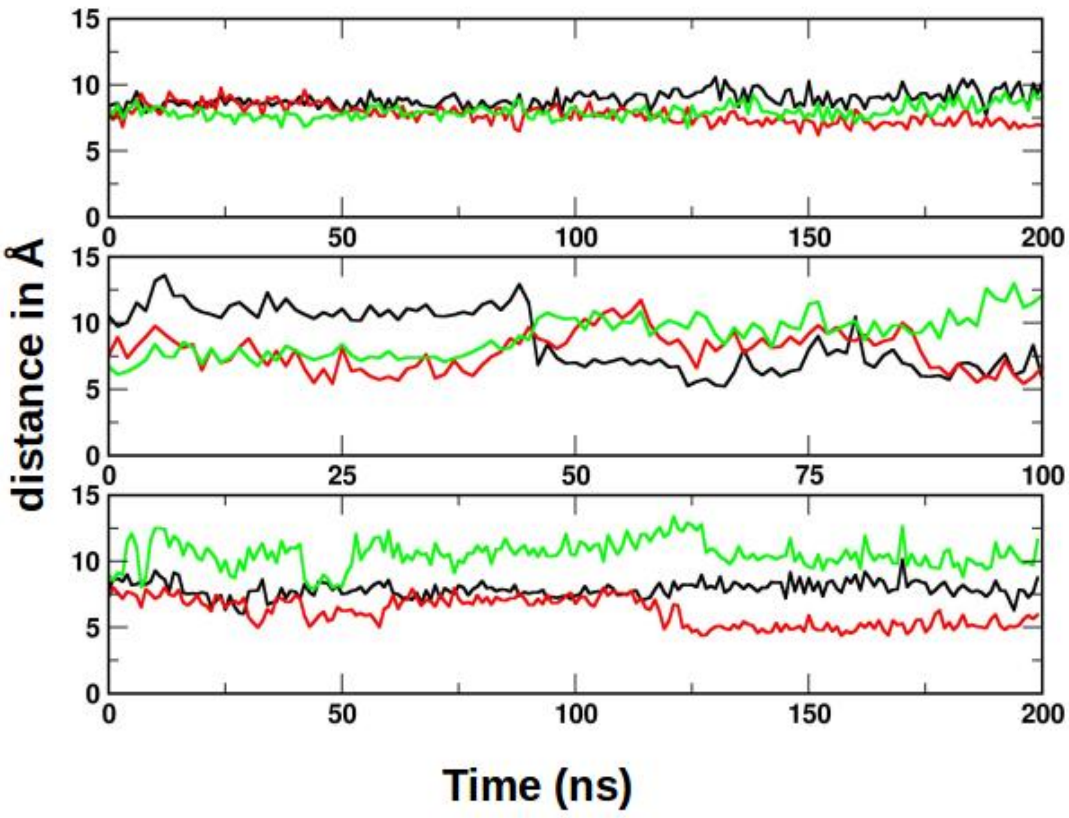

Supplementary Figure S13. Distance between the Q head group (atom C3) and Cδ atom of Glu223 (black), Glu225 (red) and Glu248 (green) from simulation setups III, VI and VII, respectively. Compared to the relatively fixed position of Q head group (see main text), amino acid residues show large scale protonation state dependent sidechain fluctuations (see also Supplementary Table S3 and Fig. S3).

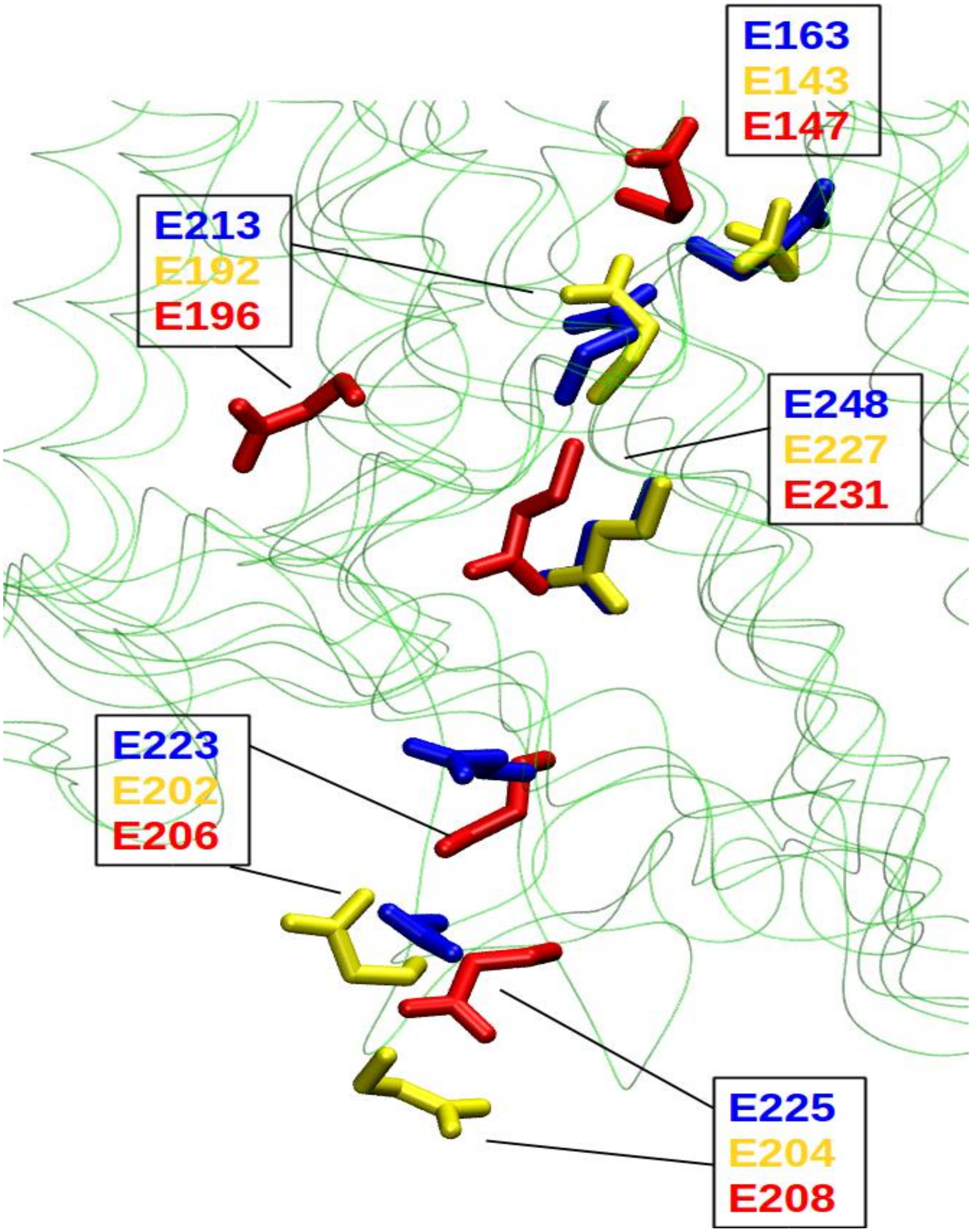

Supplementary Figure S14. Conformational variations in the sidechains or backbone of acidic amino acid residues in crystal or cryo-EM structures of complex I from *Thermus thermophilus* (blue), *Yarrowia lipolytica* (red) and *Ovis aries* (yellow). Subunits (corresponding to Nqo8 from *T. thermophilus*) are shown in green ribbon representations.

Supplementary Table S1. Residue numbers used to calculate the water occupancy in Fig. 2, and their conservancy in the *E. coli* enzyme. Subunit nomenclature of complex I from other organisms is also shown.

Subunits (*T. thermophilus* nomenclature)	Subunits (*E. coli* nomenclature)	Subunits (*B. taurus* nomenclature)	Subunits (*Y. lipolytica* nomenclature)	Residues*	Regions**
Nqo12	NuoL	ND5	NU5M	**S138** (S150)	*Closer to the N-side*
				E132 *(E144)*<br>**R163** *(R175)*<br>**D166** *(D178)*<br>***K216*** *(K229)*	*Closer to the Nqo13 subunit*
				H241 *(H254)*<br>**S301** (S314)<br>**H321** *(H334)*<br>**K329** (K342)<br>**T413** *(T425)*<br>**Y416** *(Y428)*	*In the middle of the subunit*
				**S384** (S398)<br>***K385*** *(K399)*<br>**D386** *(D400)*	*Closer to the P-side*
Nqo13	NuoM	ND4	NU4M	**H218** (H248)<br>**N221** (A251)	*Closer to the N-side*
				E123 *(E144)*<br>**S156** (G182)<br>***K204*** *(K234)*	*Closer to the Nqo14 subunit*
				**H211** (H241)<br>**T232** (I262)	*In the middle of*

				K235 *(K265)*<br>**H292** *(H322)*<br>**S318** *(H348)*<br>**Y405** *(Y435)*	*the subunit*
				E377 *(E407)*	*Closer to the Nqo12 subunit*
				**S302** *(T332)*<br>**T380** (I410)	*Closer to the P-side*
Nqo14	NuoN	ND2	NU2M	**E131** (E154)<br>**K135** (K159)<br>**D198** (D229)	*Closer to the N-side*
				E112 *(E133)*<br>***K186*** *(K217)*	*Closer to Nqo7/10/11 subunits*
				**H193** (H224)<br>***K216*** *(K247)*<br>**Y260** (Y300)<br>**S261** *(S301)*<br>**Y288** *(Y333)*	*in the middle of the subunit*
				K345 *(K395)*<br>**Y374** *(Y424)*<br>**Y375** *(Y425)*	*Closer to the Nqo13 subunit*
				**Q279** (E324)<br>**Y284** (Y329)	*Closer to the P-side*
Nqo7	NuoA	ND3	NU3M	**D72** (D79)<br>**E74** *(E81)*	
Nqo10	NuoJ	ND5	NU6M	**N35** (S35)	

				**Q55** *(E55)*<br>**Y59** *(Y59)*	
Nqo11	NuoK	ND4L	NULM	**E67** *(E72)*<br>**E32** *(E36)*<br>**N36** *(N40)*<br>**Q54** *(Q59)*<br>**R47** *(S51)*	
Nqo8	NuoH	ND1	NU1M	**E35** (E36)<br>**E130** (G124)<br>**E163** (E157)<br>**E213** (V206)<br>**E248** (E241)	
Nqo4	NuoD	49 kDa	NUCM	**H34** (H220)<br>**H38** (H224)<br>**Y87** (Y273)<br>**D139** (D329)	

[*] The residue numbers shown in bold font correspond to the *T. thermophilus* enzyme, whereas the residues marked in parentheses are from *E. coli* enzyme. Residue numbers displayed in italics correspond to those amino acids in Nqo12-14 that form the putative proton channels based on crystal structure data of *Thermus* enzyme (PDB id 4HEA), whereas *E. coli* complex I residues in italicized font are the ones found in the simulation work by Kaila et al. (see reference 31 and main text) **.**

[**] The selected residues in each of the antiporter-like subunits have been further split into different regions for additional analysis (see also Fig. S2, and main text).

Supplementary Table S2. Calculated pK_as of selected amino acid residues (see also Methods). Average pK_a values along with the standard deviation are given. In setups I and II all amino acid residues of Nqo12-14 were simulated in their standard protonation states. Protonation states of acidic residues of Nqo8 and Nqo7 subunits were altered in setups VI-X. Representative data from setups VI, VII and IX is shown (see also Fig. S3).

	**Setup I**	**Setup II**		**Setup VI**	**Setup VII**	**Setup IX**
Nqo12			Nqo8			
R163	14.27 ± 0.71	16.30 ± 0.88	E35	3.68 ± 0.36	5.76 ± 1.30	5.10 ± 0.56
D166	4.11 ± 1.30	5.96 ± 0.56	E130	6.40 ± 0.60	6.12 ± 0.61	6.27 ± 0.90
D386	5.19 ± 0.44	5.89 ± 0.44	E163	7.50 ± 0.65	7.40 ± 0.45	10.82 ± 2.88
E132	5.60 ± 0.90	5.10 ± 1.18	E213	6.70 ± 0.93	5.25 ± 0.51	8.24 ± 1.70
H241	2.93 ± 0.30	2.38 ± 1.00	E223	6.76 ± 0.98	0.91 ± 0.59	1.49 ± 0.46
H321	2.37 ± 0.37	2.94 ± 0.38	E225	3.10 ± 1.20	4.30 ± 0.91	4.16 ± 0.51
K216	12.28 ± 0.51	9.44 ± 0.20	E248	8.67 ± 1.17	7.46 ± 0.89	5.02 ± 0.79
K329	5.71 ± 0.20	6.56 ± 0.44	Nqo7			
K385	9.94 ± 0.29	7.97 ± 0.55	D72	6.19 ± 0.31	6.37 ± 0.32	7.11 ± 0.40
Y416	11.89 ± 0.39	12.61 ± 0.44	E74	7.29 ± 0.30	6.59 ± 0.55	8.24 ± 0.26
	**Setup I**	**Setup II**		**Setup I**	**Setup II**	
Nqo13			Nqo14			
E123	4.40 ± 0.45	4.52 ± 0.49	D198	3.02 ± 0.75	3.65 ± 0.55	
E377	5.31 ± 0.62	5.80 ± 0.75	E112	4.77 ± 0.57	4.92 ± 0.44	
H211	3.06 ± 1.10	4.79 ± 1.09	E131	4.75 ± 0.47	4.54 ± 0.53	
H218	4.21 ± 0.81	4.04 ± 0.79	H193	3.75 ± 0.24	3.73 ± 0.31	
H292	3.82 ± 0.59	3.42 ± 0.25	K135	10.98 ± 0.25	10.98 ± 0.29	
K204	8.87 ± 0.43	8.84 ± 0.60	K186	11.37 ± 0.98	9.73 ± 0.63	
K235	7.46 ± 0.38	7.20 ± 0.38	K216	7.02 ± 0.84	6.59 ± 0.76	
Y405	12.91 ± 0.65	13.01 ± 0.59	K345	6.78 ± 0.23	6.95 ± 0.26	
			Y260	13.12 ± 1.29	14.57 ± 0.68	
			Y284	15.56 ± 0.57	15.24 ± 0.53	
			Y288	12.96 ± 0.77	12.82 ± 1.39	
			Y374	15.93 ± 1.82	12.95 ± 2.33	
			Y375	14.33 ± 1.93	14.39 ± 0.86	

Supplementary Table S3. Root mean square fluctuation (RMSF) of the sidechains of amino acid residues in different simulations.*

simulation setup	E225 *Nqo8*	E223 *Nqo8*	E248 *Nqo8*	E35 *Nqo8*	E213 *Nqo8*	E163 *Nqo8*	D72 *Nqo7*	E74 *Nqo7*
III	1.09	1.04	1.29	0.85	1.78	1.76	1.06	1.26
IV	1.29	1.32	1.74	1.26	**1.47**	1.19	1.29	2.04
V	**3.56**	**2.62**	1.17	1.11	1.40	1.08	1.50	1.08
VI	3.24	**3.24**	**1.63**	1.14	1.53	1.37	1.37	1.21
VII	2.29	1.19	**2.02**	**1.66**	1.44	1.24	1.36	1.45
VIII	1.84	1.18	**1.71**	**1.39**	**1.96**	1.08	1.28	1.86
IX	1.20	1.17	1.16	1.28	**1.41**	**1.41**	**1.06**	**1.08**
X	2.14	1.13	**1.49**	1.29	**1.36**	**1.21**	**1.38**	**1.19**

* RMSF of the entire sidechain is calculated by summing the RMSF of all sidechain atoms divided by the number of atoms. Prior to RMSF calculations, each simulation trajectory was aligned to the zeroth frame based on the Cα atoms of the entire protein. The values in bold correspond to protonated residues.
